# Systematic literature review of the humanistic and economic burden of focal epilepsy and primary generalized tonic–clonic seizures in adults

**DOI:** 10.1002/epi4.13011

**Published:** 2024-09-18

**Authors:** Simona Boccaletti, Eleanor Lucas, Annabel Nixon, Nikolina Boskovic, Giorgio Di Dato

**Affiliations:** ^1^ Angelini Pharma Rome Italy; ^2^ OPEN Health Bethesda Maryland USA; ^3^ Chilli Consultancy Salisbury UK

**Keywords:** epilepsy, health‐related quality of life, indirect cost

## Abstract

This systematic literature review (SLR) assessed the humanistic and economic burden of focal epilepsy and primary generalized tonic–clonic seizures (PGTCS) in adults to evaluate these domains in both populations and identify evidence gaps to inform future research. A search was conducted on December 7, 2022, using MEDLINE and Embase to identify studies published from 2012 onwards reporting humanistic burden (patient‐reported or caregiver‐reported outcomes or utilities, qualitative evaluations), economic burden (productivity loss, caregiver and societal costs of epilepsy), and sleep‐related outcomes. Of the 2830 citations identified, 136 were included. Most studies were in the focal epilepsy population; very few studies reported outcomes in the PGTCS population. The presence of epilepsy‐specific instruments varied based on the domain evaluated. Epilepsy exerted considerable humanistic and economic burden. Indicators of poor disease control (e.g., high seizure frequency, resistance to anti‐seizure medications, polypharmacy) increased epilepsy burden. Seizure frequency and type, disease severity, and polypharmacy also affected work productivity. Adults with epilepsy, particularly focal epilepsy, reported higher indirect costs, more sick days accrued, and early entry into retirement. Caregivers similarly reported high productivity loss and absenteeism related to caregiving duties. The results of this SLR highlight the high humanistic and economic burden of focal epilepsy and PGTCS, although limited data were available for the PGTCS population. The results include patient‐reported outcome data specific to focal epilepsy and PGTCS, expanding the limited humanistic burden evidence identified in previous reviews, and show the effect of poor disease control on individuals' lives and as a driver of indirect costs.

**Plain Language Summary:**

Our systematic literature review identified studies that evaluated the impact of focal epilepsy and primary generalized tonic–clonic seizures on patients and their caregivers. We found that focal epilepsy negatively impacted patients' mental health and sleep and was associated with higher indirect costs and lower work productivity in people with more severe disease. The impact of primary generalized tonic–clonic seizures on patients was rarely reported, and future research is needed.

**Key points**

- This review assessed the humanistic and economic burden of focal epilepsy and primary generalized tonic–clonic seizures.
- People with epilepsy had greater anxiety, depression, and functional impairment and poorer sleep outcomes than people without epilepsy.
- Poor disease control drove indirect costs in these epilepsy subtypes and was associated with decreased work productivity.

## 1 INTRODUCTION

Epilepsy affects more than 45 million people globally^1^ and contributes to 5% of the overall disease burden (i.e., disability‐adjusted life years) of neurological disorders worldwide.^2^ It is a complex disorder that affects patients' psychological health, independence, emotional adjustment, and employment.^3^ In people with epilepsy, focal seizures are more common than generalized tonic–clonic seizures (GTCS); although data estimating the prevalence of epilepsy by seizure type are limited, it is estimated that up to two‐thirds of the overall epilepsy population has focal epilepsy.^4^, ^5^, ^6^ Among patients with idiopathic (genetic) generalized epilepsy, 74% have primary GTCS (PGTCS) as one of their seizure types.^7^

Clinical outcomes assessments (COAs) can be used to evaluate the various aspects of a person's life that are adversely affected by a disease or condition.^8^ They have the following attributes: COAs are dependent on patient active involvement or rater judgment; can be rated by the patient or an observer; and measure a meaningful aspect of how the patient feels or functions.^8^ Types of COAs include patient‐reported outcome (PRO), clinician‐reported outcome, and caregiver‐reported outcome assessments.^8^ There are a wide range of meaningful health aspects related to focal epilepsy or PGTCS that can be explored using COAs to capture a fuller picture of the humanistic burden of these diseases. For example, seizure frequency, comorbidities, and depressive symptoms are the strongest predictors of health‐related quality of life (HRQOL) in adults with epilepsy.^9^

The direct costs of epilepsy have been estimated to be high (approximately $28 billion per year).^10^ However, indirect economic costs, such as loss of work productivity, employment status, and caregiver burden, which are all directly rated to HRQOL among those with epilepsy, are more difficult to quantify.

Although the humanistic and economic burden of epilepsy more broadly has been studied extensively, little information is available for specific epilepsy subtypes such as focal epilepsy and PGTCS. A previous targeted literature review found limited evidence on the humanistic and economic burden of focal seizures, but reported focal seizures are associated with premature death, high comorbidity, and seizure‐related injuries, as well as higher costs in adults with drug‐resistant focal seizures.^5^ To our knowledge, no literature reviews have been conducted yet in the PGTCS population.

The objective of this systematic literature review (SLR) was to assess the humanistic and economic burden (focused on indirect costs) of focal epilepsy and PGTCS for adult patients and/or their caregivers.

## 2 METHODS

### 2.1 Search strategy and study selection

Embase and MEDLINE were searched on December 7, 2022, via ProQuest. Search strings are reported in Table S1. Conference abstracts indexed in Embase were also searched via ProQuest. All citations underwent two rounds of review via two independent reviewers: (1) a title and abstract screening and (2) a full‐text screening. Any discrepancy was resolved by consensus; if agreement was unable to be reached, a third reviewer provided adjudication. The SLR was conducted according to PRISMA guidelines and was registered in the international prospective register of systematic reviews (PROSPERO) under the registration number CRD42023389036.^11^

The SLR included studies in which at least half of the study population had either focal epilepsy or PGTCS in order to capture relevant data on populations of interest even where the study was not exclusively in focal epilepsy or PGTCS (Table S2). The eligibility criteria included studies in pediatric patients and caregivers, but this paper presents results for adult populations only. The outcomes of interest were humanistic burden, defined as PROs or caregiver‐reported outcomes or utilities and qualitative evaluations, and economic burden, defined as productivity loss and caregiver and societal costs associated with the epilepsy types of interest. The use of COAs has been explored in reviews or systematic reviews in other types of epilepsy^12^ as well as other diseases.^13^, ^14^, ^15^, ^16^

Only studies published in the English language from 2012 onwards were included. An additional exclusion criterion was applied in which only studies with a sample size of 70 or greater were included (provided all other inclusion criteria were met) to increase the generalizability of the review's results and better evaluate humanistic and economic burden in the populations of interest while reducing uncertainty from small sample sizes. Implementing a sample size cutoff has been used before in reviews of humanistic outcomes in large disease areas.^17^, ^18^, ^19^ This criterion was not applied to qualitative studies, which tend to have smaller sample sizes.

### 2.2 Data extraction

Relevant data were extracted from retained studies via dual extraction. This included study information (e.g., setting, country, dates), population characteristics (e.g., sex, age), and baseline, endpoint, and change‐from‐baseline values as well as mean differences between cohorts if reported. Benchmark PRO results were reported in order of the proximal impact of epilepsy (symptom burden due to epilepsy or anti‐seizure medication [ASM], functional status, mental health, sleep, personality and beliefs, family and social support) extending out to overall HRQOL, and key qualitative study results were summarized under appropriate headings (management of disease, activities of daily living, symptoms, and other humanistic burden results).

### 2.3 Assessment of study quality

Risk of bias was assessed by two independent reviewers for retained studies except conference abstracts, which have insufficient methodological data to assess study quality. The risk of bias for any randomized trial reporting HRQOL data was assessed using the Cochrane risk‐of‐bias assessment tool for randomized trials.^20^ For all other studies, the risk of bias was assessed using the Joanna Briggs Institute (JBI) critical appraisal tool for the applicable study type (case control, cohort, cross sectional, or qualitative research).^21^

## 3 RESULTS

### 3.1 Study selection and characteristics

**FIGURE 1:**
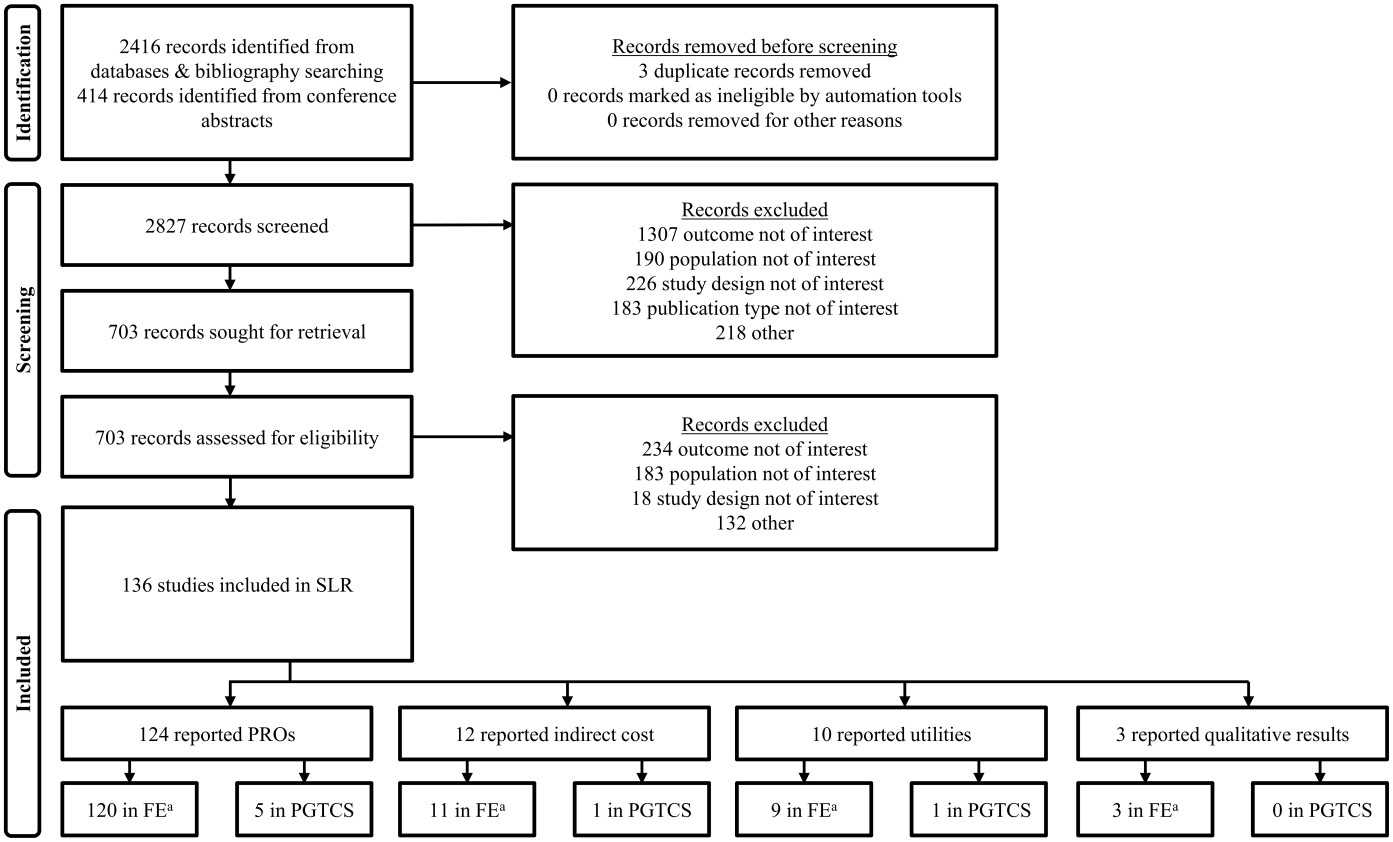
PRISMA diagram. PGTCS, primary generalized tonic–clonic seizures; PRO, patient‐reported outcome; SLR, systematic literature review. Please note that studies are non‐exclusive between topics and populations of interest and sum to >136. ^a^Includes studies where people with focal epilepsy comprised the majority of population.

A total of 2830 citations were identified (Figure 1). Of these, 136 citations were selected for inclusion into the SLR. The most common reasons for study exclusion were that the outcome, population, or study design were not of interest.

Most of the included studies reported patient‐reported outcomes. Most studies evaluated populations in the Asia Pacific Region (39 studies) and Europe (38 studies), followed by North America (29 studies; primarily those including the United States [US; 27 studies]). Studies tended to use a cross‐sectional design (96 studies), and most utilized data from 2012 onwards (68 studies).

Sample sizes ranged from nine to 1611 individuals. Studies included patients with more recently diagnosed epilepsy (minimum 4.9 years mean disease duration) to those diagnosed with epilepsy for decades (maximum 33.6 years mean disease duration). Studies included populations with a spectrum of seizure frequency, from one or two seizures per month to more than 30 seizures per month. Both monotherapy (approximately 1 mean ASM) and polytherapy (>1 ASM) populations were represented.

Risk of bias was high in one randomized controlled trial,^22^ low in one trial,^23^ with some concerns in the remaining five trials. For other study designs, appraisal via the JBI checklists did not raise any concerns regarding bias.

### 3.2 Focal epilepsy

#### 3.2.1 Humanistic burden

##### Patient‐reported outcomes

###### Symptom burden due to epilepsy or anti‐seizure medications

Four instruments evaluating the symptom burden associated with focal epilepsy and ASMs were identified: three epilepsy‐specific instruments and one generic instrument (Table S3). The instruments most frequently used to evaluate symptom burden were the epilepsy‐specific Liverpool Adverse Events Profile (LAEP; eight studies) and Liverpool Seizure Severity Scale (LSSS; four studies).

Burden due to symptoms of epilepsy or ASMs had an impact on the lives of people with epilepsy (Table 1). The mean (SD) LSSS total score among people with temporal lobe epilepsy (TLE) ranged from 39.9 (6.9) in people who were unimpaired via the Symptom Checklist‐90‐Revised to 45.6 (9.6) in people with marked symptomatology.^24^ Mean (SD) LAEP total scores were significantly lower, indicating lower burden due to adverse events (AEs), in people with focal epilepsy who had epilepsy‐related injuries or accidents than those who did not (39.2 [10.9] vs. 44.6 [9.3]; *p* = 0.007).^25^ People with TLE with poor adherence to ASMs had significantly higher mean (SD) LAEP AE scores compared to those with high‐moderate adherence (40.88 [10.19] vs. 35.99 [10.44]; *p* = 0.042).^26^

**TABLE 1:** Epilepsy‐specific patient‐ and caregiver‐reported outcome instruments measuring symptom burden due to epilepsy or anti‐seizure medications in focal epilepsy.

Instrument	References	Population (*N*)	Mean score (SD)	Comparator	*p*‐Value
LAEP	Duarte 2021^45^	People with FE (138)	Median (IQR): 33 (24–41)	NR	NA
	João 2022^28^	People with FE, caregivers with anxiety (31)	Median (range): 43 (19–68)	Caregivers without anxiety	0.22
		People with FE, caregivers without anxiety (74)	Median (range): 37 (19–76)	Reference	NA
		People with FE, caregivers with depression (29)	Median (range): 44.5 (21–68)	Caregivers without depression	0.026
		People with FE, caregivers without depression (81)	Median (range): 37 (19–76)	Reference	NA
		People with FE, caregivers with depression plus anxiety (16)	Median (range): 45.5 (26–68)	Caregivers without depression plus anxiety	0.038
		People with FE, caregivers without depression plus anxiety (84)	Median (range): 36.5 (19–76)	Reference	NA
	Lee 2014^91^	People with epilepsy (702)	34 (12.4, range: 21–81)	NR	NA
	Wang 2017^26^	People with TLE, poor adherence to ASMs (41)	40.08 (10.19)	High‐moderate adherence	0.042
		People with TLE, high‐moderate adherence to ASMs (82)	35.99 (10.04)	Reference	NA
	Willems 2018^25^	People with epilepsy (292)	39.9 (10.8)	NR	NR
		People with epilepsy, epilepsy‐related injuries and accidents (41)	39.2 (10.9)	No epilepsy‐related injuries and accidents	0.007
		People with epilepsy, no epilepsy‐related injuries and accidents (251)	44.6 (9.3)	Reference	NA
LSSS	Hamedi‐Shahraki 2020^92^	People with epilepsy (766)	54.9 (23.5)	NR	NA
	Hermann 2021^24^	People with TLE, unimpaired (39)	39.9 (6.9)	NR	NA
		People with TLE, mild‐to‐moderate symptomatology (33)	44.6 (8.6)	NR	NA
		People with TLE, marked symptomatology (22)	45.6 (9.6)	NR	NA
	Sheikh 2020^70^	People with epilepsy (379)	Mean (SD): 28.9 (29.6) Median (IQR): 25 (0–57.5)	NR	NA
		People with epilepsy, GTCS (192)	Mean (SD): 34.0 (33.3) Median (IQR): 36.25 (0–65)	No GTC seizures	0.025
		People with epilepsy, no GTCS (187)	Mean (SD): 23.7 (24.2) Median (IQR): 20 (0–45)	Reference	NA
SUDEP‐7	Dede 2019^27^	People with epilepsy, low risk of sleep apnea syndrome (114)	1.8 (1.2)	Reference	NA
		People with epilepsy, high risk of sleep apnea syndrome (25)	2.2 (1.4)	Low risk of sleep apnea syndrome	0.174

Abbreviations: ASM, anti‐seizure medication; FE, focal epilepsy; IQR, interquartile range; LAEP, Liverpool Adverse Events Profile; LSSS, Liverpool Seizure Severity Scale; NA, not applicable; NR, not reported; SCL‐90‐R, Symptom Checklist‐90‐Revised; SUDEP‐7, Sudden Unexplained Death Risk in Epilepsy; TLE, temporal lobe epilepsy.

Burden may be exacerbated by poor sleep outcomes in patients or worsening mental health outcomes in patients and/or their caregivers. People with high risk of sleep apnea syndrome had higher mean (SD) scores on the Sudden Unexplained Death Risk in Epilepsy inventory (SUDEP‐7), indicating greater risk of sudden unexplained death, compared to people with low risk of sleep apnea syndrome (2.2 [1.4] vs. 1.8 [1.2]; *p* = 0.174), though the difference was not found to be significant (Table 1).^27^ Rates of restless leg syndrome measured via the International Restless Legs Syndrome Study Group severity scale were lower in people with focal epilepsy than those with generalized epilepsy (6.5% vs. 28.6%), though the difference was also not found to be significant (*p* = 0.43).^27^ Median LAEP total scores were significantly higher, indicating greater prevalence of AEs, in people with focal epilepsy whose caregivers had depression (*p* = 0.026) or depression and anxiety (*p* = 0.038) than those without; however, there was no significant difference in scores between people with focal epilepsy whose caregivers had anxiety and those with caregivers without anxiety (*p* = 0.22).^28^

###### Functional status

Nine instruments evaluating function in people with focal epilepsy were identified: one epilepsy‐specific instrument and eight generic instruments (Table S3). The most frequently used instrument was the Self‐Report Social Adjustment Scale (SAS; two studies).

Patients with focal epilepsy indicated some confidence in their ability to manage their disease, reporting a mean (SD) score of 106.1 (32.5) out of a maximum score of 180 on a modified Korean version of the Epilepsy Self‐Efficacy Scale.^29^

Women with epilepsy had significantly lower mean scores (indicating worse sexual function) than women without epilepsy in all Female Sexual Function Index domains except pain, though a significantly larger proportion of women with epilepsy reported a score indicating sexual pain disorder.^30^ Women with epilepsy also had significantly higher mean (SD) scores on the Difficulties in Emotion Regulation Scale than men with epilepsy, indicating more difficulty with emotional regulation (80.1 [30.1] vs. 64.3 [20.5]; *p* = 0.002).^31^

Mental health disorders and previous traumatic experiences with seizures were associated with further impairment.^25^, ^32^, ^33^ People with focal epilepsy and depression (defined as an Neurological Disorders Depression Inventory for Epilepsy [NDDI‐E] score >15) had significantly higher mean (SD) scores on the Sheehan Disability Scale, indicating higher functional impairment, compared with non‐depressed patients (19.1 [8.9] vs. 9.6 [8.4]; *p* < 0.01).^32^ People with epilepsy‐related injuries and accidents had significantly higher mean (SD; range) A‐B Neuropsychological Assessment Schedule scores, suggesting greater symptomatology, than those without epilepsy‐related injuries and accidents (35.8 [17.9; 1–72] vs. 22.2 [15.7; 0–63]; corrected *p* = 0.002).^25^ People with focal epilepsy and at least one traumatic experienced seizure had significantly higher mean (SD) Dissociative Experiences Scale scores, indicating higher levels of dissociation, compared with those without traumatic experienced seizure (13.29 [8.06] vs. 7.18 [7.7]; *p* < 0.001).^33^

###### Mental health

Twenty‐two instruments evaluating mental health in people with focal epilepsy were identified, only one of which was epilepsy‐specific (Table S3). The instruments most frequently used to evaluate mental health were the Beck Depression Inventory (BDI; 29 studies), NDDI‐E (26 studies), Hospital Anxiety and Depression Scale (HADS; 22 studies), and General Anxiety Disorder‐7 (GAD‐7; 13 studies). Mean and median total scores are presented in Table 2.

**TABLE 2:** Patient‐ and caregiver‐reported outcome instruments measuring mental health in focal epilepsy.

Instrument	References	Population (*N*)	Mean score (SD)	Comparator	*p*‐Value
Epilepsy‐specific					
NDDI	Azuma 2014^93^	People with epilepsy (102)	11.7 (4.4); range: 6–23	NR	NA
	Jasionis 2021^78^	People with ETLE (29)	Median (range): 12 (6–20)	Across groups	0.133
		People with GGE (27)	Median (range): 9 (6–20)	Across groups	0.133
		People with TLE (25)	Median (range): 11 (6–24)	Across groups	0.133
	Kwon 2016^54^	People with epilepsy (276)	10.1 (4.5); range: 6–24	NR	NA
	LaFrance 2012^50^	People with intractable epilepsy, duration 0–10 years (20)	Median (range): 13.58 (10.31–18.09)	Controlled epilepsy	0.881
		People with intractable epilepsy, duration 10–20 years (23)	Median (range): 16.17 (13.16–19.84)	Controlled epilepsy	<0.0001
		People with intractable epilepsy, duration 20–30 years (36)	Median (range): 10.2 (9.11–11.53)	Controlled epilepsy	0.274
		People with intractable epilepsy, duration 30–40 years (25)	Median (range): 10.31 (8.57–12.39)	Controlled epilepsy	0.616
		People with intractable epilepsy, duration 40–50 years (32)	Median (range): 9.5 (7.20–12.39)	Controlled epilepsy	0.437
		People with intractable epilepsy, duration >50 years (16)	Median (range): 7.43 (4.88–11.30)	Controlled epilepsy	0.391
		People with controlled epilepsy, duration 0–10 years (70)	Median (range): 13.45 (10.20–17.72)	Reference	NA
		People with controlled epilepsy, duration 10–20 years (68)	Median (range): 11.18 (9.59–13.11)	Reference	NA
		People with controlled epilepsy, duration 20–30 years (52)	Median (range): 9.4 (8.48–10.41)	Reference	NA
		People with controlled epilepsy, duration 30–40 years (46)	Median (range): 9.89 (8.48–11.42)	Reference	NA
		People with controlled epilepsy, duration 40–50 years (22)	Median (range): 10.2 (7.74–13.58)	Reference	NA
		People with controlled epilepsy, duration >50 years (21)	Median (range): 6.71 (4.45–10.10)	Reference	NA
	Lee 2014^91^	People with epilepsy (702)	9.7 (4.1; range: 6–24)	NR	NA
	Malyshev 2020^49^	People with drug‐resistant FE, no seizures in previous month (NR)	Median (IQR): 9.2 (6.0–12.0)	≥1 seizure in previous month	<0.01
		People with drug‐resistant FE, ≥1 seizure in previous month (NR)	Median (IQR): 12.6 (6.0–21.0)	Reference	NA
	Moon 2016^35^	People with epilepsy (260)	10.0 (4.3; range: 6–24)	Control	<0.05
		People with epilepsy, well‐controlled (139)	8.9 (3.3; range: 6–19)	Control	NS
		People with epilepsy, poorly controlled (77)	10.2 (4.4; range: 6–24)	Control	<0.05
		People with epilepsy, uncontrolled (44)	13.2 (5.5; range: 6–24)	Control	<0.01
		Non‐epilepsy controls (200)	8.8 (3.0; range: 6–19)	Reference	NA
	Mula 2016^94^	People with epilepsy, France (116)	12.8 (4.8; range: 6–23)	NR	NA
		People with epilepsy, Germany (144)	12.7 (4.1; range: 6–24)	NR	NA
		People with epilepsy, Italy (120)	11.3 (4.3; range: 6–22)	NR	NA
	Tan 2021^95^	People with epilepsy (129)	10.39 (3.59)	NR	NA
		People with epilepsy, women (62)	10.82 (3.99)	NR	NA
		People with epilepsy, men (67)	9.99 (3.15)	NR	NA
	Tedrus 2022^96^	People with epilepsy (98)	13.3 (5.1)	NR	NA
	Villanueva 2013^48^	People with FE, drug‐resistant (184)	12.7 (4.2)	Drug‐responsive	0.023
		People with FE, drug‐responsive (53)	11.2 (3.4)	Reference	NA
Generic					
BAI	Basaran 2021^34^	People with TLE (50)	26.3 (17.6)	Controls	<0.01
		People with ETLE (51)	20.84 (16.49)	Controls	<0.01
		Non‐epilepsy controls (70)	6.24 (4.94)	Reference	NA
	João 2022^28^	People with epilepsy, clinical anxiety (492)	Median (range): 9 (0–58)	Caregivers	0.13
		People with epilepsy, concurrent clinical depression and anxiety (457)	Median (range): 25 (14–56)	Caregivers	0.56
		People with FE, clinical anxiety (376)	Median (range): 10 (0–58)	Across clinical anxiety	0.3
		People with GGE, clinical anxiety (64)	Median (range): 7 (0–50)	Across clinical anxiety	0.3
		Caregivers, clinical anxiety (168)	Median (range):7 (0–51)	Across clinical anxiety	0.3
		People with FE, concurrent clinical depression and anxiety (346)	Median (range): 25 (14–56)	Across concurrent clinical depression and anxiety	0.26
		People with GGE, concurrent clinical depression and anxiety (60)	Median (range): 30 (17–50)	Across concurrent clinical depression and anxiety	0.26
		Caregivers, concurrent clinical depression and anxiety (160)	Median (range): 29 (15–51)	Across concurrent clinical depression and anxiety	0.26
		People with epilepsy, clinical anxiety, and recurrent seizures (292)	Median (range): 11 (0–58)	Across clinical anxiety and seizure frequency	<0.001
		People with epilepsy, clinical anxiety, and fluctuating seizures (71)	Median (range): 8 (0–54)	Across clinical anxiety and seizure frequency	<0.001
		People with epilepsy, clinical anxiety, and seizure‐free (125)	Median (range): 5 (0–48)	Across clinical anxiety and seizure frequency	<0.001
		People with epilepsy, concurrent clinical depression and anxiety, and recurrent seizures (270)	Median (range): 26 (14–56)	Across concurrent clinical depression and anxiety and seizure frequency	0.35
		People with epilepsy, concurrent clinical depression and anxiety, and fluctuating seizures (67)	Median (range): 25 (14–54)	Across concurrent clinical depression and anxiety and seizure frequency	0.35
		People with epilepsy, concurrent clinical depression and anxiety, and seizure‐free (116)	Median (range): 23 (14–48)	Across concurrent clinical depression and anxiety and seizure frequency	0.35
		People with epilepsy with caregivers with depression (29)	Median (range): 18 (1–47)	Reference	NA
		People with epilepsy with caregivers without depression (81)	Median (range): 8 (0–50)	Caregivers with depression	0.015
		People with epilepsy with caregivers with anxiety (31)	Median (range): 14 (0–39)	Reference	NA
		People with epilepsy with caregivers without anxiety (74)	Median (range): 8 (0–50)	Caregivers with anxiety	0.18
		People with epilepsy with caregivers with depression plus anxiety (16)	Median (range): 17 (1–39)	Reference	NA
		People with epilepsy with caregivers without depression plus anxiety (84)	Median (range): 8.5 (0–50)	Caregivers with depression plus anxiety	0.06
	Nogueira 2017^47^	People with MTLE with negative psychiatric symptoms (61)	Median (range): 0 (0–8)	NR	NR
		People with MTLE with subsyndromic forms of depressive episodes and/or subsyndromic forms of anxiety episodes (26)	Median (range): 5.5 (0–13)	NR	NR
		People with MTLE with mood disorders or anxiety disorders (25)	Median (range): 4 (0–18)	NR	NR
		People with MTLE with mixed mood disorders/anxiety disorders (32)	Median (range): 12 (2–30)	NR	NR
		People with MTLE, pharmacoresistant disease (82)	Median (range): 5 (0–30)	Treatment‐responsive	<0.01
		People with MTLE, treatment‐responsive disease (62)	Median (range): 2 (0–26)	Reference	NA
BDI I	Basaran 2021^34^	People with ETLE (51)	18.72 (13.23)	Controls	<0.01
		People with TLE (50)	23.7 (15.3)	Controls	<0.01
		Non‐epilepsy controls (70)	5.08 (3.73)	Reference	NA
	Bosak 2015^97^	People with epilepsy (301)	Total score: 11.5 (9.7)	NR	NR
		People with epilepsy, depressed (84)	Total score: 20.5 (7.9)	Non‐depressed	<0.001
		People with epilepsy, non‐depressed (205)	Total score: 7.8 (7.7)	Reference	NA
	Cavalcanti 2019^98^	People with MTLE, unilateral hippocampal sclerosis, and depression (28)	17 (8.56)	Without depression	0.001
		People with MTLE, unilateral hippocampal sclerosis without depression (71)	10.7 (7.45)	Reference	NA
	Foldvary‐Schaefer 2012^99^	People with epilepsy (130)	10.5 (8)	NR	NR
	Grigg‐Damberger 2020^100^	People with epilepsy (99)	Median (IQR): 8 (4.0, 16.0)	NR	NR
		People with epilepsy, Mean Sleep Latency ≥8 group (56)	Median (IQR): 10 (3.5, 14.5)	Mean Sleep Latency <8	0.97
		People with epilepsy, Mean Sleep Latency <8 group (43)	Median (IQR): 8 (4.0, 17.0)	Reference	NA
		People with epilepsy, Mean Sleep Latency ≥5 group (73)	Median (IQR): 8 (3.0, 15.0)	Mean Sleep Latency <5	0.25
		People with epilepsy, Mean Sleep Latency <5 group (26)	Median (IQR): 10 (5.0, 17.0)	Reference	NA
	Han 2015^62^	People with epilepsy (NR)	16.3 (11.1)	NR	NR
		Caregivers (NR)	13.7 (11.1)	NR	NR
	Helmstaedter 2012^101^	People with TLE or FLE, mesial pathology (NR)	12 (9)	Without mesial pathology	0.028
		People with TLE or FLE, no mesial pathology (NR)	10 (8)	Reference	NA
	Lee 2022^43^	People with epilepsy (316)	16.6 (11.2; range: 0–63)	NR	NR
		People with epilepsy, men (171)	16.5 (11.7)	Women	NS
		People with epilepsy, women (145)	16.7 (10.6)	Reference	NA
		People with epilepsy, middle school or lower education (68)	21.2 (12.2)	High school or higher education	<0.001
		People with epilepsy, high school or higher education (248)	15.3 (10.6)	Reference	NA
		People with epilepsy, unemployed (92)	21.1 (12.4)	Employed	<0.001
		People with epilepsy, employed (224)	14.7 (10.2)	Reference	NA
		People with epilepsy, married (152)	17.6 (12.1)	Unmarried	NS
		People with epilepsy, unmarried (164)	15.7 (10.2)	Reference	NA
		People with epilepsy, seizures ≥1/month (87)	18.8 (10.8)	Across seizure frequency	<0.05
		People with epilepsy, seizures 1–11/year (142)	16.7 (12.1)	Across seizure frequency	<0.05
		People with epilepsy, seizure‐free (87)	14.2 (9.73)	Across seizure frequency	<0.05
		People with epilepsy, ASM polytherapy (197)	18.5 (12.1)	No ASM polytherapy	<0.001
		People with epilepsy, no ASM polytherapy (119)	13.5 (8.8)	Reference	NA
		People with epilepsy, GTCS or FBTCS in previous year (125)	19.2 (11.8)	No GTCS or FBTCS in previous year	<0.01
		People with epilepsy, no GTCS or FBTCS in previous year (191)	14.9 (10.5)	Ref	NA
		People with epilepsy, felt stigma (109)	23.1 (12.2)	No felt stigma	<0.001
		People with epilepsy, no felt stigma (207)	13.2 (9.6)	Reference	NA
	Lee 2023^46^	People with epilepsy (299)	16.5 (11.2)	NR	NR
		People with epilepsy, age <40 years (NR)	15.3 (9.3)	Age ≥40 years	<0.1
		People with epilepsy, age ≥40 years (136)	17.9 (13.1)	Reference	NA
		People with epilepsy, male (157)	16.3 (11.7)	Female	NS
		People with epilepsy, female (NR)	16.7 (10.7)	Reference	NA
		People with epilepsy, middle school or lower education (65)	20.9 (12.4)	Higher than middle school education	<0.01
		People with epilepsy, higher than middle school education (234)	15.3 (10.6)	Reference	NA
		People with epilepsy, unemployed (88)	20.7 (12.5)	Employed	<0.001
		People with epilepsy, employed (NR)	14.7 (10.2)	Reference	NA
		People with epilepsy, unmarried (NR)	15.4 (10.2)	Married	<0.1
		People with epilepsy, married (144)	17.6 (12.2)	Reference	NA
		People with epilepsy, seizure frequency: ≥1/month (83)	18.7 (10.7)	Across seizure frequency	<0.05
		People with epilepsy, seizure frequency: 1–11/year (135)	16.6 (12.2)	Across seizure frequency	<0.05
		People with epilepsy, seizure frequency: seizure‐free (81)	14 (9.6)	Across seizure frequency	<0.05
		People with epilepsy, ASM polytherapy (190)	18.6 (12.1)	No ASM polytherapy	<0.001
		People with epilepsy, no ASM polytherapy (NR)	12.8 (8.3)	Reference	NA
		People with epilepsy, GTCS or FBTCS in the past year (112)	19 (12)	No GTCS or FBTCS in the past year	<0.01
		People with epilepsy, no GTCS or FBTCS in the past year (NR)	14.9 (10.4)	Reference	NA
	Lee 2020^59^	People with epilepsy (273)	16 (11)	NR	NR
		People with epilepsy, SSE Score 0 (Total Group) (176)	12.6 (9.4)	Score ≥1 (Total Group)	<0.001
		People with epilepsy, SSE Score ≥1 (Total Group) (97)	22.2 (11)	Reference	NA
		People with epilepsy, SSE Score 0 (Enacted stigma) (19)	17.9 (7.7)	Score ≥1 (Enacted stigma)	<0.1
		People with epilepsy, SSE Score ≥1 (Enacted stigma) (46)	24.6 (12.3)	Reference	NA
		People with epilepsy, SSE Score 0 (No Enacted stigma) (157)	12 (9.4)	Score ≥1 (Enacted stigma)	<0.001
		People with epilepsy, SSE Score ≥1 (No Enacted stigma) (51)	20.1 (9.2)	Reference	NA
	Nogueira 2017^47^	People with MTLE with negative psychiatric symptoms (61)	Median (range): 2 (0–7)	NR	NR
		People with MTLE with subsyndromic forms of depressive episodes and/or subsyndromic forms of anxiety episodes (26)	Median (range): 12 (1–16)	NR	NR
		People with MTLE with mood disorders or anxiety disorders (25)	Median (range): 14 (3–24)	NR	NR
		People with MTLE with mixed mood disorders/anxiety disorders (32)	Median (range): 27 (13–41)	NR	NR
		People with MTLE, pharmacoresistant disease (82)	Median (range): 12 (0–41)	Treatment‐responsive group	<0.01
		People with MTLE, treatment‐responsive disease (62)	Median (range): 4 (0–37)	Reference	NA
BDI‐II	Abe 2020^102^	People with epilepsy (113)	12 (10)	People with psychogenic nonepileptic spells	<0.01
		People with psychogenic nonepileptic spells (36)	19 (10)	Reference	NA
		People with TLE (65)	12 (10)	Reference	NA
		People with ETLE (28)	11 (7)	TLE	0.4
	Bosak 2015^103^	People with epilepsy (289)	11.53 (9.68)	Reference	NA
		People with epilepsy, depression (121)	20.2 (6.94)	People with epilepsy	<0.05
		People with epilepsy, recent suicide ideation (30)	26 (7.82)	NR	NR
	Bosak 2016^104^	People with epilepsy, no suicidal ideation (271)	10.4 (8.6)	Reference	NA
		People with epilepsy, suicidal ideation (30)	25.2 (9.1)	No suicidal ideation	<0.001
	Garcia 2015^51^	People with FE (515)	Adjusted^a^, 50.5 (95% CI: 46.2–54.8)	NR	NR
		People with FE, controlled disease (267)	Adjusted^a^, 37.2 (95% CI: 31.4–43.0)	NR	NR
		People with FE, drug‐resistant disease (248)	Adjusted^a^, 64.8 (95% CI: 58.8–70.8)	NR	NR
	Hermann 2021^24^	People with TLE, unimpaired (39)	4.4 (4.6)	Controls	NS
		People with TLE, mild‐to‐moderate symptomatology (33)	8.1 (4.7)	Controls	0.022
		People with TLE, marked symptomatology (22)	18.6 (8.1)	Controls	<0.001
		Non‐epilepsy controls (94)	4.7 (5.1)	Reference	NA
	João 2022^28^	People with epilepsy, clinical depression (497)	Median (range): 11 (0–57)	Caregivers	0.007
		People with epilepsy, concurrent clinical depression and anxiety (457)	Median (range): 25 (14–57)	Caregivers	0.65
		People with FE, clinical depression (372)	Median (range): 11 (0–57)	Across clinical depression	0.018
		People with GGE, clinical depression (69)	Median (range): 9 (0–49)	Across clinical depression	0.018
		Caregivers, clinical depression (174)	Median (range): 7 (0–56)	Across clinical depression	0.018
		People with FE, concurrent clinical depression and anxiety (346)	Median (range): 24 (14–57)	Across concurrent clinical depression and anxiety	0.45
		People with GGE, concurrent clinical depression and anxiety (60)	Median (range): 30 (14–49)	Across concurrent clinical depression and anxiety	0.45
		Caregivers, concurrent clinical depression and anxiety (160)	Median (range): 24 (14–56)	Across concurrent clinical depression and anxiety	0.45
		People with epilepsy, clinical depression, and recurrent seizures (295)	Median (range): 13 (0–57)	Across clinical depression and seizure frequency	<0.001
		People with epilepsy, clinical depression, and fluctuating seizures (74)	Median (range): 11 (0–41)	Across clinical depression and seizure frequency	<0.001
		People with epilepsy, clinical depression, and seizure‐free (124)	Median (range): 6 (0–47)	Across clinical depression and seizure frequency	<0.001
		People with epilepsy, concurrent clinical depression and anxiety, and recurrent seizures (270)	Median (range): 26 (14–57)	Across concurrent clinical depression and anxiety and seizure frequency	0.37
		People with epilepsy, concurrent clinical depression and anxiety, and fluctuating seizures (67)	Median (range): 21 (14–41)	Across concurrent clinical depression and anxiety and seizure frequency	0.37
		People with epilepsy, concurrent clinical depression and anxiety, and seizure‐free (116)	Median (range): 23 (14–47)	Across concurrent clinical depression and anxiety and seizure frequency	0.37
		People with epilepsy with caregivers with depression (29)	Median (range): 22 (3–46)	Reference	NA
BDI‐II	
		People with epilepsy with caregivers without depression (81)	Median (range): 9 (0–41)	Caregivers with depression	<0.001
		People with epilepsy with caregivers with anxiety (31)	Median (range): 16 (0–36)	Reference	NA
		People with epilepsy with caregivers without anxiety (74)	Median (range): 9 (0–41)	Caregivers with anxiety	0.041
		People with epilepsy with caregivers with depression plus anxiety (16)	Median (range): 22.5 (3–36)	Reference	NA
	People with epilepsy with caregivers without depression plus anxiety (84)	Median (range): 8 (0–46)	Caregivers with depression plus anxiety	0.007
	Lozano‐García 2021^105^	People with drug‐resistant TLE (75)	12.72 (8.52); range: 0.00–34.90	NR	NR
	Tan 2021^95^	People with epilepsy, men, English BDI‐II (25)	9.6 (8.69)	NR	NR
		People with epilepsy, women, English BDI‐II (25)	9.08 (7.37)	NR	NR
		People with epilepsy, men, Malay BDI‐II (42)	8 (8.51)	NR	NR
		People with epilepsy, women, Malay BDI‐II (37)	9.84 (7.99)	NR	NR
		People with epilepsy, English BDI‐II (50)	9.38 (7.98)	NR	NR
		People with epilepsy, Malay BDI‐II (79)	8.86 (8.3)	NR	NR
	Tombini 2021^31^	People with epilepsy, female (72)	12.3 (10.5)	Male population	0.035
People with epilepsy, male (50)		9.3 (7.4)	Reference	NA
GAD‐7	Abe 2020^102^	People with epilepsy (113)	9 (7)	People with psychogenic nonepileptic spells	0.04
		People with psychogenic nonepileptic spells (36)	12 (5)	Reference	NA
		People with TLE (65)	9 (6)	Reference	NA
		People with ETLE (28)	10 (8)	TLE	0.2
	Duarte 2021^45^	People with epilepsy (138)	Median (IQR): 4 (2–6)	NR	NR
	Kwon 2016^54^	People with epilepsy (276)	4.8 (5.4); range: 0–21	NR	NR
	Lee 2022^41^	People with epilepsy (146)	4.1 (4.4)	NR	NR
	Lee 2014^91^	People with epilepsy (702)	4.1 (4.9); range: 0–21	NR	NR
	Moon 2016^35^	People with epilepsy (260)	4.6 (5.2); range: 0–21	Control	NS
		People with epilepsy, well‐controlled disease (139)	3 (3.7); range: 0–16	Control	NS
		People with epilepsy, poorly controlled disease (77)	5.4 (5.9); range: 0–21	Control	<0.05
		People with epilepsy, uncontrolled disease (44)	8.4 (6); range: 0–21	Control	<0.01
		Control (200)	3.5 (3.7); range: 0–19	Reference	NA
	Sheikh 2020^70^	People with epilepsy (379)	Mean (SD): 8.5 (6.4) Median (IQR): 7 (3–13)	NR	NR
		People with epilepsy, GTCS (192)	Mean (SD): 9.5 (6.5) Median (IQR): 9 (4.5–14)	No GTC seizures	0.049
		People with epilepsy, no GTCS (187)	Mean (SD): 7.6 (6.3) Median (IQR): 7 (2–12)	Reference	NA
		People with epilepsy, GTCS, 0 seizures in past month (85)	Mean (SD): 7.9 (6.2) Median (IQR): 7 (4–12)	Across seizure frequency	0.347
		People with epilepsy, GTCS, 1–4 seizures in past month (69)	Mean (SD): 10.2 (6.4) Median (IQR): 9 (4.75–15)	Across seizure frequency	0.347
		People with epilepsy, GTCS, ≥5 seizures in past month (38)	Mean (SD): 10.6 (6.7) Median (IQR): 10 (5–15)	Across seizure frequency	0.347
HADS total	Lee 2020^29^	People with epilepsy (312)	12.6 (7.3)	NR	NR
	Rigon 2019^106^	People with epilepsy (100)	13.6 (7.7)	Reference	NA
		Non‐epilepsy controls (50)	12.6 (6.6)	People with epilepsy	0.422
		People with TLE (63)	13.5 (7.9)	Non‐TLE	0.776
		People with epilepsy other than TLE (37)	13.9 (7.6)	Reference	NA
HADS anxiety	Lee 2023^107^	People with refractory focal seizures (85)	7.5 (4.4)		
		People with refractory focal seizures, males (49)	7.6 (4.5)	Reference	NA
		People with refractory focal seizures, females (36)	7.3 (4.4)	Males	0.712
HADS depression	Dabla 2020^108^	People with epilepsy (100)	7.77 (3.25)	NR	NR
	Duarte 2021^45^	People with epilepsy (138)	Median (IQR): 6 (3–9)	NR	NR
	Lee 2023^107^	People with refractory focal seizures (85)	7.6 (4.1)	NR	NR
		People with refractory focal seizures, males (49)	7.6 (4.5)	Reference	NA
		People with refractory focal seizures, females (36)	7.3 (4.4)	Males	0.662
	Lee 2021^109^	People with epilepsy (357)	5.95 (3.74)	NR	NR
	Shin 2022^110^	People with epilepsy (129)	Median (IQR): 3 (0.0–6.0)	NR	NR
MADRS	Garcia 2015^51^	People with FE (515)	Adjusted^a^, 46.8 (95% CI: 42.5–51.1)	NR	NR
		People with FE, controlled disease (267)	Adjusted^a^, 32.6 (95% CI: 27.0–38.2)	NR	NR
		People with FE, drug‐resistant disease (248)	Adjusted^a^, 62.1 (95% CI: 56.0–68.2)	NR	NR
PHQ‐9	Abe 2020^102^	People with epilepsy (113)	8 (6)	People with psychogenic nonepileptic spells	<0.01
		People with psychogenic nonepileptic spells (36)	11 (5)	Reference	NA
		People with TLE (65)	8 (6)	Reference	NA
		People with ETLE (28)	7 (6)	TLE	0.22
	Im 2016^38^	People with epilepsy (180)	6.2 (5.5)	Control	<0.001
		Non‐epilepsy controls (2836)	2.3 (3.5)	Reference	NA
	Lee 2022^41^	People with epilepsy (146)	6.0 (5.8) (5.8)	NR	NR
	Lu 2021^111^	People with epilepsy (148)	5.9 (5.4)	NR	NR
	Sheikh 2020^70^	People with epilepsy (379)	Mean (SD): 8.5 (6.5) Median (IQR): 7 (3–13)	NR	NR
		People with epilepsy, GTCS (192)	Mean (SD): 9.5 (6.5) Median (IQR): 9 (4.5–14)	No GTCS	0.035
		People with epilepsy, no GTCS (187)	Mean (SD): 7.6 (6.3) Median (IQR): 7 (2–12)	Reference	NA
		People with epilepsy, GTCS, 0 seizures in past month (85)	Mean (SD): 7.9 (6.2) Median (IQR): 7 (4–12)	Across seizure frequency	0.347
		People with epilepsy, GTCS, 1–4 seizures in past month (69)	Mean (SD): 10.2 (6.4) Median (IQR): 9 (4.75–15)	Across seizure frequency	0.347
		People with epilepsy, GTCS, ≥5 seizures in past month (38)	Mean (SD): 10.6 (6.7) Median (IQR): 10 (5–15)	Across seizure frequency	0.347
PSS	Lee 2022^43^	People with epilepsy reporting feelings of stigma (109)	20.1 (4.5)	NR	NR
	Moon 2016^35^	People with epilepsy (260)	16.8 (6.4; range: 0–35)	Controls	NS
		Non‐epilepsy controls (200)	16.2 (5.4; range: 0–29)	Reference	NA
		People with epilepsy, well‐controlled (139)	14.6 (5.8; range: 0–28)	Controls	<0.01
		People with epilepsy, poorly controlled (77)	17.7 (5.8; range: 5–29)	Controls	NS
		People with epilepsy, uncontrolled (44)	21.9 (5.9; range: 11–35)	Controls	<0.01
STAI – state anxiety	Ertan 2021^42^	People with FE, drug‐resistant disease with anticipatory anxiety of epileptic seizure (46)	41.4 (12.3)	No anticipatory anxiety of epileptic seizure	0.07
		People with FE, drug‐resistant disease without anticipatory anxiety of epileptic seizure (41)	33.6 (13.3)	Reference	NA
STAI – trait anxiety	Ertan 2021^42^	People with FE, drug‐resistant disease with anticipatory anxiety of epileptic seizure (46)	46.6 (12.5)	No anticipatory anxiety of epileptic seizure	0.02
		People with FE, drug‐resistant disease without anticipatory anxiety of epileptic seizure (41)	39.9 (11.6)	Reference	NA
	Lozano‐Garcia 2021^105^	People with FE (75)	26.08 (10.68); range: 0.00–49.00	NR	NR

Abbreviations: ASM, anti‐seizure medication; BAI, Beck Anxiety Inventory; BDI, Beck Depression Inventory; ETLE, extratemporal lobe epilepsy; FBTCS, focal bilateral tonic–clonic seizures; FE, focal epilepsy; GAD‐7, General Anxiety Disorder 7‐item; GGE, idiopathic (genetic) generalized epilepsy; GTCS, generalized tonic–clonic seizures; HADS, Hospital Anxiety and Depression Scale; JME, juvenile myoclonic epilepsy; MADRS, Montgomery‐Åsberg Depression Rating Scale; MTLE, mesial temporal lobe epilepsy; NA, not applicable; NDDI‐E, Neurological Disorder Depression Inventory for Epilepsy; NR, not reported; NS, not significant; PHQ‐9, Patient Health Questionnaire 9‐Item; PSS, Perceived Stress Scale; SCL‐90‐R, Symptom Checklist‐90‐Revised; SSE, Stigma Scale of Epilepsy; STAI, State and Trait Anxiety Scale; TLE, temporal lobe epilepsy.

^a^ Adjusted for patients on antidepressant treatment.

Depression and anxiety were the most frequently evaluated patient‐reported outcomes in people with epilepsy and were routinely found to be significantly worse in this population compared with non‐epilepsy controls (Table 2). Mean Beck Anxiety Inventory (BAI),^34^ GAD‐7,^35^ State Trait Anxiety Scale (STAI),^36^, ^37^ and Zung Self‐Rating Anxiety Scale^30^ scores were significantly higher (indicating greater anxiety) in people with any epilepsy or poorly controlled epilepsy compared with non‐epilepsy controls. People with any epilepsy or poorly controlled epilepsy had significantly higher BDI^24^, ^34^, ^36^ and Patient Health Questionnaire 9‐Item (PHQ‐9)^38^ scores (indicating more severe depression) than non‐epilepsy controls.

Large proportions of patients met clinical criteria for anxiety or depression, and scores for anxiety and/or depression were commonly above cutoffs for pathological symptoms.^33^, ^38^, ^39^, ^40^, ^41^, ^42^ The proportion of people with depression (as defined by each study) ranged from 16%^25^ to 68%.^43^ Rates of moderate depression (PHQ‐9 score ≥10) ranged from 19%^38^ to 19.9%^41^ in South Korean studies and 16.9% of people in a US study had moderately severe depression (PHQ‐9 score ≥15).^40^ In a Turkish cross‐sectional study, 35% of people reported anxiety and/or depression as measured by the BAI and BDI.^44^ In a Brazilian cross‐sectional study, 56% of people with epilepsy reported generalized anxiety to be moderately to extremely disabling.^45^ In a Chinese cross‐sectional study, people with TLE with poor medication adherence reported more moderate–severe anxiety via the BAI than people with high‐moderate adherence.^26^

Characteristics associated with poor disease control, such as higher seizure frequency,^43^, ^46^ recurrent seizures,^35^ and drug‐resistance and polytherapy,^43^, ^46^, ^47^, ^48^ were associated with additional mental health impairment in populations with epilepsy. Significant differences in BAI or BDI scores were found by seizure frequency,^28^, ^43^, ^46^ polytherapy,^43^, ^46^ and pharmacoresistance (Table 2).^47^ NDDI‐E scores were significantly higher (indicating higher prevalence of depressive symptoms) in people with drug‐resistant epilepsy^47^, ^48^ or poorly controlled/uncontrolled seizures.^35^, ^49^, ^50^ Montgomery‐Åsberg Depression Rating Scale scores were also higher (indicating worse depressive symptoms) in people with drug‐resistant focal epilepsy than those with well‐controlled focal epilepsy (statistical significance not tested).^51^ Anxiety as measured by the BAI was significantly higher in people with pharmacoresistant mesial TLE than treatment‐responsive mesial TLE (*p* < 0.01)^47^ and in people with epilepsy with recurrent seizures and clinical anxiety than those who had clinical anxiety who were seizure‐free (*p* < 0.001).^28^ In a French cohort study, a GAD‐7 score of 8 or greater was more common in patients with pharmacoresistant focal epilepsy with versus without at least one traumatic experienced seizure (*p* = 0.08), though not significantly so.^33^

###### Sleep

Eight instruments evaluating sleep in people with focal epilepsy were identified: all were generic instruments (Table S3). The most frequently used instruments evaluating sleep were the Epworth Sleepiness Scale (11 studies) and Pittsburgh Sleep Quality Index (six studies).

In some studies, people with focal epilepsy had worse sleep quality than people with generalized epilepsy,^52^ while in others, no difference was observed.^27^ Some studies showed no difference in outcomes between adults with epilepsy and non‐epilepsy controls^53^; however, for outcomes such as excessive daytime sleepiness, insomnia, and sleep quality, adults with epilepsy had worse outcomes than non‐epilepsy controls^38^, ^53^ and a larger proportion of adults with epilepsy met pathological criteria for the disorder than controls.^38^, ^53^

Poor sleep outcomes tended to be more prevalent with characteristics that indicated poor disease control, such as drug‐resistant epilepsy^39^ or more frequent seizures,^27^ though this was not found in all studies.

###### Personality and beliefs

Thirteen instruments evaluating function in people with focal epilepsy were identified: one epilepsy‐specific instrument and 12 generic instruments (Table S3). The most frequently used instruments for evaluating personality and beliefs were the Buss‐Perry Aggression Questionnaire (two studies) and Rosenberg Self‐Esteem Scale (two studies).

Irritability levels were generally low‐to‐moderate in people with epilepsy, though they were higher in people with poorly controlled and uncontrolled epilepsy.^54^ ASM treatment in people with drug‐refractory focal epilepsy did lead to an improvement in irritability over short‐term follow‐up.^55^ General self‐efficacy was lower in people with ASM polytherapy than those with no polytherapy,^46^ and self‐esteem and work motivation were diminished in people with low employability versus high employability.^56^ Scores on the Buss‐Perry Aggression Questionnaire were significantly higher in people with epilepsy and depression than non‐epilepsy controls with idiopathic major depression.^57^

###### Family and social support

Eight instruments evaluating family and social support in people with focal epilepsy were identified: all were generic instruments (Table S3). The most frequently used instrument evaluating family and social support was the Family Adaptability, Partnership, Growth, Affection, and Resolve Scale (APGAR, three studies). Measures of family functioning, such as the APGAR, found at least mild familial dysfunction in over half of people with epilepsy and their caregivers. Familial support and satisfaction were further negatively affected by the presence of mental health illness and disorders such as depression^58^ and perceived stigma^59^ as well as with low adherence to ASMs.^26^ The presence of depression was also tied to low levels of social support in adults with epilepsy.^60^

###### Stigma and discrimination

Six instruments evaluating stigma and discrimination in people with focal epilepsy were identified: two epilepsy‐specific instruments and four generic instruments (Table S3). The most frequently used instruments evaluating stigma and discrimination were the Stigma Scale of Epilepsy (SSE; five studies) and Epilepsy Stigma Scale (four studies).

Over one‐third (35.5%) of people with epilepsy perceived stigma via the SSE,^59^ while 48.9% perceived stigma (30% reporting high perceived stigma) via the Epilepsy Stigma Scale.^61^ Perceived stigma was experienced similarly by both people with epilepsy and their caregivers.^62^ Almost one quarter (23.8%) of people with epilepsy reported having felt enacted stigma (Table 2).^59^ Stigma was associated with family and social support and employment; with increasing perceptions of stigma associated with worse family functioning or unemployment/low employability.^56^, ^59^, ^63^

###### HRQOL

Ten instruments evaluating HRQOL in people with focal epilepsy were identified, of which four were epilepsy‐specific (Table S3). The most frequently used instruments were iterations of the Quality of Life in Epilepsy Inventory (QOLIE‐31: 40 studies; QOLIE‐10: 12 studies; QOLIE‐31‐P: eight studies) and the EQ‐5D (nine studies). Overall HRQOL in people with focal epilepsy was lower than non‐epilepsy controls (Table 3).^34^, ^64^ HRQOL was also worse, often significantly so, in people with epilepsy and psychiatric disorders, particularly depression, than in those without psychiatric disorders.^58^, ^60^, ^65^

**TABLE 3:** Patient‐ and caregiver‐reported outcome instruments measuring overall HRQOL in focal epilepsy.

Instrument	Reference	Population (*N*)	Mean (SD) score	Comparator	*p*‐Value
Epilepsy specific					
ESI‐55	Rayner 2016^58^	People with epilepsy, cognitive depression (15)	45.00 (13.37, range: 25–75)	NR	NR
		People with epilepsy, somatic depression (6)	53.00 (16.14, range: 50–77.5)	NR	NR
		People with epilepsy, no depression (70)	63.639 (14.727, range: 27.5–100)	NR	NR
QOLIE‐89 total score	Hermann 2021^24^	People with TLE, unimpaired (39)	56.2 (5.2)	Between groups	<0.01
		People with TLE, mild‐to‐moderate symptomatology (33)	48.5 (6.9)	Between groups	<0.01
		People with TLE and marked symptomatology (22)	39.5 (7.97)	Between groups	<0.01
	Munger Clary 2018^112^	People with FE, lower anxiety (540)	51.9 (10.2)	High anxiety	<0.001
		People with FE, higher anxiety (540)	40.8 (10.5)	Reference	NA
QOLIE‐31 total score	Abe 2019^113^	People with TLE (65)	56 (16)	People with ETLE	0.352
		People with ETLE (28)	58 (19)	Reference	NA
	Andrade‐Machado 2015^88^	People with FE, refractory disease (82)	53.09 (18.29; range: 14.02–89.4)	NR	NA
		People with refractory FE, without risk of suicide (33)	57.8 (16.9, range: 26–89)	People with refractory FE and risk of suicide	<0.05
		People with refractory FE, with risk of suicide (49)	46.0 (18.2, range: 14–88)	Reference	NA
	Cano‐López 2022^114^ ^,^ ^a^	People with TLE, drug‐resistant disease (119)	36.91 (27.22)	NR	NA
	Elsharkawy 2012^115^	People with refractory FE, no surgery (125)	54.5 (NR)	NR	NA
		Patients with refractory FE, no surgery, perceived seizure status as “improved significantly” (19)	67.0 (21.9)	Perceived seizure status as “improved”	<0.05
		Patients with refractory FE, no surgery, perceived seizure status as “improved” (35)	55.5 (16.5)	Reference	NA
		Patients with refractory FE, no surgery, reported “very good” AED tolerability (24)	65.6 (13.4)	Reported “unsatisfactory”	<0.001
		Patients with refractory FE, no surgery, reported “unsatisfactory” AED tolerability (10)	41.1 (11.8)	Reference	NA
	Ertan 2021^42^	People with anticipatory anxiety of epileptic seizure (46)	34.5 (21.7)	People without anticipatory anxiety of epileptic seizure	0.2
		People without anticipatory anxiety of epileptic seizure (41)	41.2 (19.7)	Reference	NA
	Garcia 2015^51^	People with FE, drug‐resistant disease (248)	54.2 (18.9)	Reference	NA
		People with FE, controlled disease (267)	69.9 (16.5)	People with drug‐resistant FE	0.001
	Hamedi‐Shahraki 2020^92^	People with epilepsy (766)	0.68 (0.2)	NR	NA
	Jasionis 2021^78^	People with ETLE (29)	61.03 (15.85)	Across groups	0.201
		People with TLE (25)	56.73 (18.61)	Across groups	0.201
		People with GGE (27)	65.78 (18.10)	Across groups	0.201
	Lee 2021^109^	People with epilepsy (357)	59 (17.2)	NR	NA
	Lozano‐García 2021^105^	People with FE (75)	52.8 (13.77; range: 22.00–87.00)	NR	NA
	Lu 2021^111^	People with epilepsy (148)	54.2 (10.1)	NR	NA
	Pauli 2012^89^	People with refractory MTLE‐HS (81)	Median (IQR): 40.8 (25.1–52.3)	NR	NA
		People with refractory MTLE‐HS, male (35)	Median (IQR): 41.8 (34.1–52.5)	Reference	NA
		People with refractory MTLE‐HS, female (46)	Median (IQR): 37.4 (22.4–52.5)	Male	0.19
		People with refractory MTLE‐HS, single (46)	Median (IQR): 42.6 (23.7–51.7)	Reference	NA
		People with refractory MTLE‐HS, married (35)	Median (IQR): 39.7 (26.1–53.8)	Married	0.89
		People with refractory MTLE‐HS, work activity (50)	Median (IQR): 40.6 (25.2–55.3)	Reference	NA
		People with refractory MTLE‐HS, no work activity (31)	Median (IQR): 41.6 (25–50.1)	Work activity	0.45
		People with refractory MTLE‐HS, no history of IPI (23)	Median (IQR): 52.1 (25.4–60.8)	Reference	NA
		People with refractory MTLE‐HS, history of IPI (52)	Median (IQR): 40.1 (25–49.4)	No history of IPI	0.08
		People with refractory MTLE‐HS, no family history of epilepsy (65)	Median (IQR): 41.8 (25.8–54.3)	Reference	NA
		People with refractory MTLE‐HS, family history of epilepsy (15)	Median (IQR): 36.8 (19.1–46.4)	No family history	0.08
		People with refractory MTLE‐HS, right MRI side (36)	Median (IQR): 42.2 (25.8–53.7)	Reference	NA
		People with refractory MTLE‐HS, left MRI side (39)	Median (IQR): 40.4 (22.7–52.1)	Right MRI side	0.61
		People with refractory MTLE‐HS, bilateral MRI (6)	Median (IQR): 35.1 (23.8–42.9)	Right MRI side	0.61
		People with refractory MTLE‐HS, no anxiety disorders (64)	Median (IQR): 42.7 (25.5–53.1)	Reference	NA
		People with refractory MTLE‐HS, anxiety disorders (17)	Median (IQR): 36.9 (25–51.5)	No anxiety disorders	0.45
		People with refractory MTLE‐HS, no depressive disorders (65)	Median (IQR): 44.6 (28.6–52.6)	Reference	NA
		People with refractory MTLE‐HS, depressive disorders (16)	Median (IQR): 36.5 (20.4–50.1)	No depressive disorders	0.07
		People with refractory MTLE‐HS, no Axis II diagnosis (63)	Median (IQR): 45.5 (28.2–54.3)	Reference	NA
		People with refractory MTLE‐HS, Axis II diagnosis (18)	Median (IQR): 28.9 (19.5–41.8)	No Axis II diagnosis	0.02
		People with refractory MTLE‐HS, monotherapy (11)	Median (IQR): 40.3 (15.9–67.8)	Reference	NA
		People with refractory MTLE‐HS, monotherapy plus benzodiazepine (24)	Median (IQR): 46.7 (29.8–55.7)	Monotherapy	0.52
		People with refractory MTLE‐HS, polytherapy (46)	Median (IQR): 39.7 (24.8–50.2)	Monotherapy	0.52
	Rigon 2019^106^ ^,^ ^a^	People with TLE (63)	58.4 (16.1)	People with epilepsy other than TLE	0.755
		People with epilepsy other than TLE (37)	59.7 (20.7)	Reference	NA
	Shin 2022^110^	People with epilepsy (80)	Median (IQR): 32.5 (25.9–42.2)	NR	NA
	Taskiran 2019^44^	People with FE (105)	57.2 (27.0)	NR	NA
		People with FE, good seizure control and no affective symptoms (38)	82.9 (14.4)	Across groups	<0.001
		People with FE, good seizure control and affective symptoms (6)	38.5 (29.8)	Across groups	<0.001
		People with FE, poor seizure control and no affective symptoms (30)	57.1 (18.3)	Across groups	<0.001
		People with FE, poor seizure control and affective symptoms (31)	29.5 (12.0)	Across groups	<0.001
	Tedrus 2015^116^	People with epilepsy (159)	58.1 (15.8)	NR	NA
	Tedrus 2016^117^	People with epilepsy (200)	65.9 (16.3)	NR	NA
		People with NDDI‐E ≤15 (117)	Median (IQR): 67.4 (54.7–75.8)	People with NDDI‐E >15	<0.01
		People with NDDI‐E >15 (19)	Median (IQR): 47.8 (32.0–66.0)	Reference	NA
	Tedrus 2018^118^	People with epilepsy (148)	60.6 (15.3)	NR	NA
	Tedrus 2022^96^	People with epilepsy (98)	55.0 (16.4)	NR	NA
	Tombini 2021^31^	People with epilepsy, childhood onset (≤18 years) (35)	59.2 (17)	Across groups	0.046
		People with epilepsy, adult‐onset (19–59 years) (64)	51.3 (18.3)	Across groups	0.046
		People with epilepsy, older age‐onset (≥60 years) (23)	60.2 (17.9)	Across groups	0.046
	Witt 2014^119^	People with new onset FE prior to treatment (233)	73.6 (13.8)	NR	NA
QOLIE‐31‐P total score	Flint 2022^64^	People with FE (361)	45.72 (14.29; range: 8.60–86.33)	NR	NA
	Unterberger 2015^120^	People with epilepsy (200)	Median (range): 83.2 (32.1–100)	NR	NA
	Villanueva 2013^48^	People with FE, drug‐resistant (184)	56.4 (18.7)	Drug‐responsive	<0.0001
		People with FE, drug‐responsive (53)	70.8 (13.3)	Reference	NA
Modified QOLIE‐31‐P total score	Fawale 2014^61^	People with epilepsy (93)	72.0 (15.1)	NR	NA
		People with epilepsy, seizure‐free (19)	83.1 (11.3)	Across seizure severity levels	0.000
		People with epilepsy, low‐moderate seizure severity (28)	71.6 (14.1)	Across seizure severity levels	0.000
		People with epilepsy, high seizure severity (46)	67.6 (15.0)	Across seizure severity levels	0.000
	Malyshev 2021^121^	People with FE (111)	Median (range): 65.4 (53–72.6)	NR	NA
QOLIE‐10 total score	Fonseca 2021^52^	People with epilepsy (142)	71.16 (18.81)	NR	NA
		People with epilepsy, ESS score >12 (28)	60 (21.8)	Mild excessive daytime sleepiness; Normal daytime sleepiness	<0.001
		People with epilepsy, ESS score 11–12 (25)	66 (16.3)	Reference	NR
		People with epilepsy, ESS score 0–10 (88)	76.6 (16.2)	Reference	NR
		People with epilepsy, HADS anxiety score ≥11 (23)	48.5 (15.5)	HADS anxiety score ≤10	<0.001
		People with epilepsy, HADS anxiety score ≤10 (124)	74.7 (16.6)	Reference	NR
		People with epilepsy, HADS depression score ≥11 (9)	45.9 (22)	HADS depression score ≤10	<0.001
		People with epilepsy, HADS depression score ≤10 (139)	72.8 (17.7)	Reference	NR
	George 2015^122^	People with partial epilepsy (116)	73.96 (20.63)	People with generalized seizures	0.621
		People with generalized seizures (84)	75.43 (20.66)	Reference	NA
	Lee 2014^91^	People with epilepsy (702)	76.1 (18.2; range: 3–100)	NR	NA
	Lee 2020^29^	People with epilepsy (312)	74.1 (19.2)	NR	NA
	Ranjana 2014^123^	People with FE or generalized epilepsy (451)	64.1 (15.97; range: 15.97–100)	NR	NA
	Salas‐Puig 2019^124^	People with FE or PGTCS, no accidental injuries due to seizures (162)	71.5 (17.2)	Reference	NA
		People with FE or PGTCS, accidental injuries due to seizures (239)	64.3 (20.7)	Without injuries	0.0003
	Sheikh 2019^125^	People with epilepsy, active GTCS (NR)	28 (NR)	Non‐GTC epilepsy	0.044
		People with epilepsy, non‐GTCS (NR)	25 (NR)	Reference	NA
Generic					
EQ‐5D VAS	Mulhern 2017^67^	People with FE, newly diagnosed (1611)	68.14 (20.7; range: 5–100)	NR	NR
	Soare 2022^66^	Caregivers (345)	64.23 (21.41)	NR	NR
	Villanueva 2013^48^	People with FE, drug‐resistant disease (184)	64.7 (19.1)	Drug‐responsive	0.0008
		People with FE, drug‐responsive disease (53)	75.6 (16.5)	Reference	NA
	Willems 2018^25^	People with epilepsy (292)	66.7 (19.3; range: 10–100)	NR	NR
		People with epilepsy, epilepsy‐related injuries and accidents (41)	56 (19.1; range: 10–90)	No epilepsy‐related injuries and accidents	<0.001
		People with epilepsy, no epilepsy‐related injuries and accidents (251)	68.4 (18.7; range: 20–100)	Reference	NA
Global QOL overall score	Endermann 2013^126^	People with epilepsy, mild intellectual disability (142)	6.14 (2.03)	NR	NR
WHOQOL‐26 total score	Suda 2016^65^	People with localization‐related epilepsy, no psychiatric disorder (57)	3.32 (0.42)	Reference	NA
		People with localization‐related epilepsy, psychiatric disorder (46)	3.09 (0.51)	Without psychiatric disorder	0.04
		People with localization‐related epilepsy, IDD (25)	2.78 (0.39)	Without psychiatric disorder	<0.01
WHOQOL‐BREF total score	Baniya 2022^60^	People with epilepsy, no depression (207)	49.63 (7.03)	Reference	NA
		People with epilepsy, depression (145)	45.74 (7.54)	Without depression	<0.05

Abbreviations: ESI‐55, Epilepsy Surgery Inventory–55 Items; ESS, Epworth Sleepiness Scale; ETLE, extratemporal lobe epilepsy; FE, focal epilepsy; GGE, idiopathic (genetic) generalized epilepsy; GTCS, generalized tonic–clonic seizures; HADS, Hospital Anxiety and Depression Scale; HRQOL, health‐related quality of life; IDD, interictal dysphoric disorder; IPI, initial precipitant injury; IQR, interquartile range; MTLE‐HS, mesial temporal lobe epilepsy related to hippocampal sclerosis; NA, not applicable; NDDI‐E, Neurological Disorders Depression Inventory for Epilepsy; NR, not reported; PGTCS, Primary generalized tonic–clonic seizures; PRO, patient‐reported outcome; QOL, quality of life; QOLIE, Quality of Life in Epilepsy Inventory; SCL‐90‐R, Symptom Checklist‐90‐Revised; TLE, temporal lobe epilepsy; VAS, visual analog scale; WHOQOL, World Health Organization Quality of Life.

^a^ Study reports total score only.

Characteristics associated with poor disease control were also associated with HRQOL impairment. The EQ‐5D visual analog scale score was significantly lower (indicating worse HRQOL) in people with drug‐resistant focal epilepsy compared with drug‐responsive focal epilepsy (*p* = 0.0008)^48^ and in people with epilepsy‐related injuries and accidents compared with people without epilepsy‐related injuries and accidents (*p* < 0.001)^25^ (Table 3). In an international cross‐sectional analysis, both mental and physical health tended to increase with better seizure control: mental and physical component summary scores via a mix of the SF‐12 version 2 and SF‐36 version 2 were significantly lower in people with at least one seizure per week compared with people with less than one seizure per year except for physical component summary scores in the US.^6^ In another international cross‐sectional study of caregivers of adults with uncontrolled drug‐resistant focal‐onset seizures, caregivers reported the greatest problems (moderate, severe, or extreme problems) in anxiety/depression (44% of caregivers) and pain/discomfort (40%).^66^

##### Health utility measures

Nine studies reported health utility values in people with focal epilepsy (Table 4). Only one epilepsy‐specific instrument, the Quality of Life in Newly Diagnosed Epilepsy Instrument, was used (one study). The most frequently used instruments for measuring health utility were the EQ‐5D‐5L (four studies), SF‐6D (two studies), and EQ‐5D‐5L (two studies). Lower utility values were seen with increasing seizure frequency.^67^, ^68^ Additionally, differences in utility values were found by seizure type (e.g., simple vs. complex),^69^ location,^6^ and response to treatment.^70^

**TABLE 4:** Health utility values in adults with focal epilepsy or PGTCS and their caregivers.

Instrument	References	Population (*N*)	Timepoint	Mean (SD) health utility value^a^
Focal epilepsy				
EQ‐5D‐3L	Mukuria 2017^127^	People with uncontrolled focal seizures enrolled in study N01252 (355)	Baseline	0.756 (0.234)
			Week 12 or 16	0.770 (0.241)
		People with uncontrolled focal seizures enrolled in study N01253 (347)	Baseline	0.762 (0.226)
			Week 12 or 16	0.791 (0.221)
		People with uncontrolled focal seizures enrolled in study N01254 (393)	Baseline	0.758 (0.234)
			Week 12 or 16	0.771 (0.229)
		Pooled analysis of three clinical trials of adults with uncontrolled focal seizures (1095)	Baseline	0.759 (0.232)
			Week 12 or 16	0.777 (0.230)
		Pooled analysis of three clinical trials of adults with uncontrolled focal seizures experiencing one to two seizures per week (485)	Baseline	0.748 (0.24)
		Pooled analysis of three clinical trials of adults with uncontrolled focal seizures experiencing two or more seizures per week (188)	Baseline	0.775 (0.21)
	Mulhern 2017^67^	People with newly diagnosed FE (1563)	Baseline	0.735 (0.30); Median (range): 0.848 (−0.38 to 1)
		People with newly diagnosed FE (1244)	Year 1	0.769 (0.29); Median (range): 0.848 (−0.454 to 1)
		People with newly diagnosed FE (1091)	Year 2	0.789 (0.28); Median (range): 0.848 (−0.239 to 1)
EQ‐5D‐5L	Hixson 2021^128^	People with focal seizures treated with eslicarbazepine acetate as first adjunctive therapy to levetiracetam or lamotrigine monotherapy (44)	Baseline	0.97 (NR)
			Week 11	0.95 (NR)
			Week 27	0.94 (NR)
		People with focal seizures treated with eslicarbazepine acetate as later adjunctive therapy (58)	Baseline	0.95 (NR)
			Week 11	0.95 (NR)
			Week 27	0.96 (NR)
	Phumart 2018^68^	People with FE (181)	NA	0.77 (NR)
		People with FE, seizure‐free (49)	NA	0.81 (NR)
		People with FE, seizure reduction (80)	NA	0.78 (NR)
		People with FE, no seizure improvement (52)	NA	0.72 (NR)
		People with FE, seizure reduction or no seizure improvement (132)	NA	0.76 (NR)
	Soare 2022^66^	Caregivers of people with uncontrolled focal‐onset seizures (345)	NA	0.6 (0.27); range: −0.24 to 1; Median (IQR): 0.62 (0.44–0.8)
	Tritton 2019^69^	People with focal simple partial seizures (92)	NA	0.450 (NR)
		People with focal complex partial seizures (83)	NA	0.454 (NR)
NEWQOL‐6D	Mulhern 2017^67^	People with newly diagnosed FE receiving carbamazepine, gabapentin, lamotrigine, oxcarbazepine, or topiramate in the SANAD study (1508)	Baseline	0.766 (0.13); Median (range): 0.786 (0.341 to 0.957)
		People with newly diagnosed FE receiving carbamazepine, gabapentin, lamotrigine, oxcarbazepine, or topiramate in the SANAD study (1156)	Year 1	0.798 (0.13); Median (range): 0.832 (0.341 to 0.957)
		People with newly diagnosed FE receiving carbamazepine, gabapentin, lamotrigine, oxcarbazepine, or topiramate in the SANAD study (1023)	Year 2	0.805 (0.13); Median (range): 0.844 (0.341 to 0.957)
		People with newly diagnosed FE, 1–3 seizures in the last year (468)	Baseline	0.8 (0.11)
		People with newly diagnosed FE, 4–9 seizures in the last year (432)	Baseline	0.77 (0.12)
		People with newly diagnosed FE, ≥10 seizures in the last year (599)	Baseline	0.74 (0.14)
SF‐6D	Flint 2022^64^	People with FE (361)	NR	0.584 (0.111); Median (range): 0.578 (0.322–0.929)
	Gupta 2017^6^	People with FE in Brazil (71)	NA	0.64 (0.11)
		People with FE in 5EU (257)	NA	0.66 (0.13)
		People with FE in 5EU + Brazil (328)	NA	0.65 (0.13)
		People with FE in the US (345)	NA	0.67 (0.14)
Standard gamble	Sheikh 2020^77^	People with DR‐TLE undergoing medical treatment, seizure‐free (NR)	NA	Mean (95% CI): 0.96 (0.84–1)
		People with DR‐TLE undergoing surgery with no major complication, seizure‐free (NR)	NA	Mean (95% CI): 0.97 (0.87–1)
		People with DR‐TLE undergoing surgery with major complications, seizure‐free (NR)	NA	Mean (95% CI): 0.77 (0.32–1)
		People with DR‐TLE experiencing seizures undergoing medical treatment (NR)	NA	Mean (95% CI): 0.75 (0.38–1)
		People with DR‐TLE experiencing seizures undergoing surgery with no major complication (NR)	NA	Mean (95% CI): 0.78 (0.41–1)
		People with DR‐TLE experiencing seizures undergoing surgery with major complication (NR)	NA	Mean (95% CI): 0.66 (0.19–1)
PGTCS				
SF‐6D	Tremblay 2018^81^	People with PGTCS, >53 seizures/year (NR)	NA	0.59 (0.14)
		People with PGTCS, 13–52 seizures/year (NR)		0.63 (0.15)
		People with PGTCS, 1–12 seizures/year (NR)		0.63 (0.13)
		People with PGTCS, seizure‐free (NR)		0.71 (0.15)

Abbreviations: DR‐TLE, drug‐resistant temporal lobe epilepsy; FE, focal epilepsy; IQR, interquartile range; NA, not applicable; NEWQOL, Quality of Life in Newly Diagnosed Epilepsy Instrument; NR, not reported; PGTCS, primary generalized tonic–clonic seizures; QWB‐SA, Quality of Well Being Self‐Administered; TTO, time trade off; US, United States; VAS, visual analog scale.

^a^ Mean (SD) unless otherwise noted.

##### Qualitative evaluation

Three qualitative studies reported the humanistic burden of people with focal epilepsy (Table S4).^71^, ^72^, ^73^ Studies evaluated mental health (anxiety, depression, and stigma), symptoms and function (disease symptoms, postictal phenomena, functional impairment, return to normal activities), and disease management (coping strategies, self‐management). A high level of burden was associated with everyday tasks and disease management. Over half of adults with focal epilepsy reported an inability to drive, limited ability to go to work and/or school, and limitations on leisure and social activities due to epilepsy.^34^ Furthermore, barriers to self‐management of disease in adults with epilepsy including focal epilepsy and PGTCS included adjusting to life with epilepsy, cognitive impairment hindering self‐management, epilepsy‐related communication difficulties, and difficulties related to managing multiple illnesses.^74^

#### 3.2.2 Economic burden

##### Indirect cost

Five studies reporting indirect costs in people with focal epilepsy were identified for the US (four studies) and Europe (two studies) (Table 5).^6^, ^66^, ^75^, ^76^, ^77^ Total annual indirect costs for individuals with epilepsy were highest in the US ($8095–$24 247) compared with individuals in the EU5 (France, Germany, Italy, Spain, and the United Kingdom; €5736–€11 178).^6^ Indirect costs were higher in people with uncontrolled seizures compared with controlled seizures,^77^ with increasing seizure frequency,^6^ and in people treated with gabapentin compared with pregabalin.^76^ Among caregivers of people with epilepsy, indirect costs were higher in employed spouses of people with partial seizures on monotherapy than employed spouses of people with partial seizures on adjunctive therapy.^75^

**TABLE 5:** Indirect cost and productivity loss among adults with focal epilepsy or PGTCS and their caregivers.

Reference	Population (*N*)	Country	Study date	Perspective	Year and currency	Measure	Economic outcome	Value
Focal epilepsy								
Brook 2018^75^	Employed spouses of adults with partial onset seizures on monotherapy (238)	US	2001–2014	NR	2017 USD ($)	PPPY	Total	$912
							Sick leave	$582 (SE $38)
							Short‐term disability	$18 (SE: $16)
							Long‐term disability	$0
							Workers' compensation	$312 (SE: $156)
							Total absence days	2.7
							Days of sick leave	2.4
							Days of short‐term disability	0.3
	Employed spouses of adults with partial onset seizures on adjunctive therapy (129)	US	2001–2014	NR	2017 USD ($)	PPPY	Total	$1192
							Sick leave	$1123 (SE: $90)
							Short‐term disability	$62 (SE: $32)
							Long‐term disability	$0
							Workers' compensation	$7 (SE: $7)
							Total absence days	5.1
							Days of sick leave	4.4
							Days of short‐term disability	0.7
	Difference between cohorts	US	2001–2014	NR	2017 USD ($)	Difference in PPPY	Sick leave	$541 (*p* = 0.001)
							Short‐term disability	$44 (*p* = 0.2200)
							Workers' compensation	−$305 (*p* = 0.0497)
							Total absence days	*p* < 0.05
							Days of sick leave	*p* < 0.0001
							Days of short‐term disability	Not significant
Gupta 2017^6^	Adults with FE (354)	US	2011–2013	Patient	2013 USD ($)	PPPY	Absenteeism	$1629.15
							Presenteeism	$6555.67
							Total indirect costs	$8095.63
							Total indirect costs (ages 18–60 years)	$24 247.30
					NA	Per year for all patients	Absenteeism^a^	6.33%
							Presenteeism^a^	24.38%
							Overall work impairment (absenteeism + presenteeism)^a^	26.82%
							Activity impairment^a^	37.25%
	Adults with FE (257)	France, Germany, Italy, Spain, UK	2011 and 2013	Patient	2013 Euro (€)	PPPY	Absenteeism	€1629.14
							Presenteeism	€4229.84
							Total indirect costs	€5736.38
							Total indirect costs (ages 18–60 years)	€11 178.69
					NA	Per year for all patients	Absenteeism^a^	7.80%
							Presenteeism^a^	26.34%
							Overall work impairment (absenteeism + presenteeism)^a^	30.68%
							Activity impairment^a^	36.34%
	Adults with FE (71)	Brazil	2012	Patient	NA	Per year for all patients	Absenteeism^a^	9.16%
							Presenteeism^a^	25.10%
							Overall work impairment (absenteeism + presenteeism)^a^	29.14%
							Activity impairment^a^	25.92%
Kleinman 2012^76^	Adults receiving gabapentin (107)	US	2004–2011	Employer	NR USD ($)	6‐month post‐index cost per cohort	Short‐term disability cost	$1280
							Sick leave cost	$552
							Workers' compensation cost	$170
					NA		Short‐term disability days^b^	9.81
							Sick leave days^c^	10.81
							Workers' compensation days^d^	0
	Adults receiving pregabalin (66)	US	2004–2011	Employer	NR USD ($)	6‐month post‐index cost per cohort	Short‐term disability cost	$580
							Sick leave cost	$342
							Workers' compensation cost	$30
							Short‐term disability days^b^	6.24
							Sick leave days^c^	1.51
							Workers' compensation days^d^	0
	Difference between cohorts	US	2004–2011	Employer	NR USD ($)	Difference in 6‐month post‐index cost per cohort	Short‐term disability cost	−$700
							Sick leave cost	−$210
							Workers' compensation cost	−$139
							Short‐term disability days^b^	−3.57
							Sick leave days^c^	−9.29
							Workers' compensation days^d^	0
Sheikh 2020^77^	People with uncontrolled seizures (NR)	US	NR	Societal	2019 USD ($)	PPPY	Reduction in labor market earnings	$23 635
							Reduction in domestic productivity	$799
	People with controlled seizures (NR)	US	NR	Societal	2019 USD ($)	PPPY	Reduction in labor market earnings	$13 342
							Per patient reduction in domestic productivity	$1482
	Care providers	US	NR	Societal	2019 USD ($)	PPPY	Mean productivity loss	$11 678
Soare 2022^66^	Caregivers of adults with uncontrolled partial onset seizures (345)	UK, Germany, France, Italy, Spain, Sweden	NR	Caregiver	NR Euro (€)	PPPY	Median annualized cost of lost productivity	€7228
							Mean annualized cost of lost productivity	€14 872
					Euro (€)	PPPW	Median cost of caregiver	€139
							Mean cost of productivity loss	€286
PGTCS								
Tremblay 2018^81^	Adults with PGTCS treated with perampanel (NR)	Spain	NA	National Health Service	2017 Euros (€)	Per patient per 33‐year time horizon	Indirect cost of productivity loss	€129 613
							Mortality (indirect friction cost)	€1089
	Adults with PGTCS treated with maintenance therapy (NR)	Spain	NA	National Health Service	2017 Euros (€)	Per patient per 33‐year time horizon	Indirect cost of productivity loss	€140 253
							Mortality (indirect friction cost)	€1213

Abbreviations: EU, European Union; FE, focal epilepsy; NA, not applicable; NR, not reported; PGTCS, primary generalized tonic–clonic seizures; PPPW, per patient per week; PPPY, per patient per year; UK, United Kingdom; US, United States; USD, United States dollar; WPAI, Work Productivity and Activity Impairment.

^a^ Derived via WPAI. Absenteeism defined as percentage of work time missed due to health. Presenteeism defined as percentage of impairment while working due to health. Activity impairment defined as percentage of impairment overall due to health.

^b^ Defined as the number of days absent during periods of short‐term disability leaves (acquired from disability insurance carrier data).

^c^ Defined as the number of days absent during sick leave (acquired through payroll data).

^d^ Defined as the number of days absent during workers' compensation leave (acquired from workers' compensation claims data).

##### Productivity loss

Three studies reporting productivity loss in people with focal epilepsy were identified for the US (two studies), Brazil (one study), and Europe (two studies) (Table 5).^6^, ^75^, ^76^ Productivity loss was higher in the EU5 and Brazil than in the US,^6^ people treated with gabapentin than pregabalin,^76^ and with increasing seizure frequency.^6^ Among caregivers of people with epilepsy, productivity loss was higher in employed spouses of people with partial seizures on monotherapy than in employed spouses of people with partial seizures on adjunctive therapy.^75^

### 3.3 PGTC seizures

#### 3.3.1 Humanistic burden

##### Patient‐reported outcomes

No studies using PRO instruments to evaluate sleep, personality and beliefs, family and social support, and stigma and discrimination were identified in the PGTCS population, and no studies using a qualitative methodology were identified.

###### Symptom burden due to epilepsy or anti‐seizure medications

Symptom burden in the GTCS population was evaluated in one study using the LSSS (Table S5). Patients with drug‐resistant epilepsy who experienced GTCS had worse LSSS scores than those who did not experience GTCS, though the difference was not significant after adjustment for covariates.^70^ Scores were improved at 1‐year follow‐up in people with GTCS after correcting for multiple testing.

###### Functional status

Function in the GTCS population was evaluated in one study that used the SAS to evaluate self‐reported social adjustment in people with juvenile myoclonic epilepsy with GTCS (Table S5).^37^ Patients experiencing GTCS had more severe social maladjustment than non‐epilepsy controls, as measured by significantly higher mean (SD) SAS scores for the total (2.14 [0.75] vs. 1.62 [0.55]; *p* < 0.01), work (2.00 [0.98] vs. 1.44 [0.5]; *p* < 0.01), social and leisure (2.07 [0.68] vs. 1.73 [0.6]; *p* = 0.02), family relationship (1.98 [0.61] vs. 1.64 [0.46]; *p* = 0.01), and economic (2.11 [1.27] vs. 1.7 [0.97]; *p* = 0.02) domains.^37^

###### Mental health

Six instruments evaluating mental health in people with GTCS were identified: one epilepsy‐specific (NDDI‐E^78^) and five generic (GAD‐7,^70^ HADS,^79^ PHQ‐9,^70^ STAI^37^; Table S5). NDDI‐E scores in people with generalized epilepsy (93% with GTCS) did not significantly differ from scores in people with (extratemporal) TLE (*p* = 0.133).^78^ PHQ‐9 scores and GAD‐7 scores were significantly higher (indicating more severe depression or anxiety) in people with GTCS than those without, though the difference was not significant after adjustment for covariates. PHQ‐9 scores were improved at 1‐year follow‐up in people with GTCS after correcting for multiple testing.^70^ In people with GTCS, significantly more people met criteria for anxiety and depression (via HADS) than non‐epilepsy controls (*p* < 0.01).^79^ Patients with GTCS had more severe state and trait anxiety than non‐epilepsy controls (*p* < 0.01).^37^

###### HRQOL

Three studies reported on HRQOL in patients with GTCS: two studies using the QOLIE‐31 instrument^78^, ^80^ and one study using the QOLIE‐10 instrument (Table S5).^70^ Mean QOLIE‐31 scores for adults with GTCS were significantly higher (indicating better HRQOL) in adults treated with monotherapy compared with polytherapy^80^; however, mean QOLIE‐31 scores did not differ between people with generalized epilepsy (93% with GTCS) and people with (extratemporal) TLE (*p* = 0.201).^78^ Mean QOLIE‐10 scores were higher in people with GTCS than those without, though the difference was not significant after adjustment for covariates; scores improved over 1 year of follow‐up after correcting for multiple testing.^70^

##### Health utility measures

One study reported health utility measures for PGTCS using the generic SF‐6D instrument (Table 4). Lower utility values were seen with increasing seizure frequency: mean (SD) utility score was 0.71 (0.15) for seizure‐free patients, 0.63 (0.13) for those with one to 12 seizures per year, 0.63 (0.15) for those with 13–52 seizures per year, and 0.59 (0.14) for those with 53 or more seizures per year.^81^

#### 3.3.2 Economic burden

No studies reporting on productivity loss in people with PGTCS were identified.

##### Indirect cost

One study reporting on indirect costs in people with PGTCS was identified for Spain.^81^ Indirect costs (€2017) per patient over a 33‐year time horizon were higher in people treated with maintenance therapy compared with perampanel for indirect costs of productivity loss (€140 253 vs. €129 613) and mortality (€1213 vs. €1089; Table 5).^6^

## 4 DISCUSSION

This review was conducted to evaluate the humanistic and economic burden of two epilepsy subtypes: focal epilepsy and PGTCS. It is the first review to include granular data on humanistic burden and indirect costs in these specific populations, including the factors that affect the humanistic burden and indirect costs and how these outcomes are measured. The included studies showed a high level of humanistic and economic burden in people with epilepsy, both when compared with non‐epilepsy populations and within epilepsy subpopulations.

Most of the included studies were in the focal epilepsy population. The SLR results highlight a substantial burden of illness in this population. Indicators of poor disease control such as high seizure frequency, resistance to ASMs, and polypharmacy were routinely found to increase the burden of focal epilepsy. Seizure frequency, seizure type, disease severity, and polypharmacy were also found to affect work productivity. Adults with focal epilepsy reported higher indirect cost, higher use of sick days, and early entry into retirement. Studies of caregivers of people with epilepsy similarly reported high productivity loss and absenteeism related to caregiving duties. These results show the continuing need to prioritize disease control to help reduce the humanistic and economic burden of focal epilepsy.

For the PGTCS population, no studies were identified using PRO instruments to evaluate sleep, personality and beliefs, family and social support, and stigma and discrimination; using a qualitative methodology; or reporting on productivity loss in people with PGTCS or their caregivers. Very few studies identified in this SLR reported outcomes in people with PGTCS; most studies identified reported on people with GTCS, primary not specified. The research gap in evidence for the PGTCS population is particularly concerning because GTCS are associated with increased risk of seizure‐related injury and sudden unexplained death in epilepsy compared with other seizure types.^82^, ^83^ The SLR results showed health utility values decreased with increasing seizure frequency in people with PGTCS, and that use of perampanel as adjunctive treatment to ASMs reduced per‐patient indirect productivity loss and mortality costs of PGTCS in Spain. People with GTCS had more severe social maladjustment than non‐epilepsy controls and had greater perceived seizure severity and more severe depression or anxiety than those without GTCS. These results highlight the need for active management of PGTCS.

The results of this SLR highlight several directions for future research. First, there is a need for more research on PGTCS‐specific outcomes. Limited economic burden, PRO, and health utility evidence was identified for adults with PGTCS, and no evidence in these domains was identified for caregivers of adults with PGTCS. More primary studies should be conducted in PGTCS. A patient survey or discrete choice experiment to evaluate economic and humanistic burden and treatment preferences in patients with PGTCS and their caregivers would be particularly impactful, as it could capture patient‐ or caregiver‐reported outcomes and other data necessary to derive inputs (e.g., indirect costs, productivity losses, health utilities) needed to inform future decision‐making tools. Second, more research is needed on the economic burden of focal epilepsy and PGTCS for adult patients and their caregivers, particularly indirect costs and productivity loss for caregivers. Future research could include database analyses to evaluate indirect costs in adults with PGTCS and caregivers of people with PGTCS or focal epilepsy and/or health economic modeling to evaluate indirect costs associated with PGTCS over the patient and caregiver lifetime from various perspectives. Such studies would also help derive indirect cost inputs needed to inform future decision‐making tools, such as cost‐utility and cost‐effectiveness analyses. Finally, future longitudinal research should explore changes in epilepsy‐associated humanistic burden and indirect costs over time. Previous SLRs on the burden of illness in epileptic disorders featuring resistant seizures, such as Lennox–Gastaut syndrome and Dravet syndrome, have also identified a notable lack of studies evaluating indirect costs and caregiver burden.^12^, ^84^ This emphasizes the need for more comprehensive research in these areas to provide a broader understanding of the economic implications and HRQOL impact in severe forms of epilepsy.

The results of this review showed that there was high variability in the presence of epilepsy‐specific PRO measures, which were more commonly used in reporting symptom burden and HRQOL versus functioning and mental health. While generic PRO measures can be used to compare patients across different health conditions, condition‐specific measures are most appropriate for measuring treatment outcomes and changes in certain aspects of health within specific clinical populations, focusing on the individual level.^85^, ^86^ Thus, there is an opportunity to develop more epilepsy‐specific PRO instruments, especially in domains such as functioning and mental health, in order to capture patient perspectives and priorities on elements of health relevant to people with epilepsy.^86^, ^87^

Strengths of our review include the inclusion of a wide range of outcomes, study designs, patient populations, and regions that may be underrepresented in literature, such as Africa and the Middle East. Additionally, when evaluating PROs, only studies with sample sizes of at least 70 people were included. Although studies with fewer than 70 people may also include relevant results, advantages of limiting to studies with larger sample sizes include greater generalizability. In addition, we did not exclude any substantial PGTCS studies that could have addressed the imbalance between focal epilepsy and PGTCS; there are simply fewer studies in the PGTCS population, most of which were captured in this SLR.

Limitations of our review included a high number of studies that were cross‐sectional in design, limiting conclusions around causal or temporal trends in the humanistic burden of focal epilepsy and PGTCS. Additionally, given differences in study designs, evaluated outcomes, and years of data collection, the generalizability of findings was limited, and comparisons across large groups of studies were restricted. Studies in people with epilepsy that did not report the population's epilepsy subtypes were excluded, even though the study may have, theoretically, included a majority of the population of interest. However, other SLRs in epilepsy have taken a similar approach and excluded mixed cohorts.^12^ As previously noted, risk of bias was high in one randomized controlled trial,^22^ low in one trial,^23^ with some concerns in the remaining five trials, but no other concerns regarding bias were identified for other study designs. The prominence of single‐center studies may have introduced selection bias into the sample.

There were also limitations associated with studies reporting humanistic burden and indirect cost of focal epilepsy and PGTCS. Several studies included patient populations specifically selected from tertiary epilepsy centers,^53^, ^88^ which may not reflect community settings. In some studies, the reported patient population was not further stratified beyond “focal” or “generalized” epilepsy (e.g., Lee 2022^41^). Some included studies were not powered to detect statistically significant changes in PROs (e.g., Pauli 2012^89^) and studies that included self‐reported patient questionnaires may have introduced response bias into the sample (e.g., Giovagnoli 2013^36^). The PRO instruments contained different recall periods, and surveys of productivity loss were subject to recall bias.^48^, ^90^ Not all PRO instruments that were used across studies were validated in the studied patient population. Finally, data around seizure types were limited in studies reporting health utility scores for people with epilepsy or their caregivers.^67^

## 5 CONCLUSIONS

This review provides insight into the high level of humanistic and economic burden associated with focal epilepsy and PGTCS in adults and their caregivers. The results include PRO data specific to the focal epilepsy and PGTCS populations and provide detailed information about the PRO instruments used, expanding the limited humanistic burden evidence identified in previous reviews. People with epilepsy had significantly greater anxiety and depression, poorer sleep outcomes, and greater functional impairment than people without epilepsy. Among people with epilepsy, poor disease control (e.g., high seizure frequency, resistance to ASMs, polypharmacy) drove indirect costs and was associated with decreased work productivity. The SLR results also highlight the complex interplay between disease control, sleep, and mental health disorders in epilepsy. However, the results also highlight the imbalanced evidence base between focal epilepsy versus PGTCS.

It is imperative that stakeholders across healthcare, research, and policy domains prioritize initiatives aimed at enhancing focal epilepsy and PGTCS management, expanding access to effective treatments, and providing comprehensive support services to mitigate the burdens faced by patients and their caregivers. This review underscores the critical need for continued research efforts to fill existing knowledge gaps and deepen our understanding of the evolving burden of PGTCS and epilepsy‐related humanistic consequences over the lifespan of affected individuals. By fostering collaborative research endeavors and driving innovation in care delivery, we can strive towards a future where the impact of epilepsy on individuals and society is significantly reduced. Elevating epilepsy as a priority on institutional agendas is essential for fostering a more just and equitable healthcare landscape.

## AUTHOR CONTRIBUTIONS

Concept and design: All authors. Collection and assembly of data: EL, NB. Data analysis and interpretation: All authors. Manuscript writing or critical review: All authors. Final approval of manuscript: All authors.

## FUNDING INFORMATION

This work was supported by Angelini Pharma. Employees of Angelini Pharma participated in the study design, interpretation of data, writing of report, and decision to submit the article for publication.

## CONFLICT OF INTEREST STATEMENT

At the time of study conduct, EL and NB were employees of OPEN Health, which received consulting fees from Angelini to conduct the review; SB and GDD were employees of Angelini. SB is currently an employee of UCB. AN is a Director at Chilli Consultancy, which received consulting fees from Angelini to participate in the review.

## ETHICAL PUBLICATION

We confirm that we have read the journal's position on issues involved in ethical publication and affirm that this report is consistent with those guidelines.

## Supporting information


Tables S1–S5.
