# Supplementary material for: Systematic literature review of the humanistic and economic burden of focal epilepsy and primary generalized tonic–clonic seizures in adults
Source: Epilepsia Open. 2024 Sep 18;9(6):2055–86. doi: 10.1002/epi4.13011 (PMC11633719; doi:10.1002/epi4.13011)
Supplement: Supplementary file 1 — Tables S1–S5. [file EPI4-9-2055-s001.docx]

# Supplemental Material

Table S1. Embase/MEDLINE Search Strategy

| **#** | **Terms** |
| --- | --- |
| S1 | TI,AB((partial OR focal OR local) NEAR/2 (epilepsy OR epileptic OR epilepsies OR seizure OR seizures)) OR EMB.EXACT.EXPLODE(“focal epilepsy”) OR MESH.EXACT.EXPLODE(“Epilepsies, Partial”) |
| S2 | TI,AB((tonic-clonic OR “grand mal”) NEAR/3 (epilepsy OR epileptic OR epilepsies OR seizure)) OR EMB.EXACT(“tonic-clonic seizure”) OR EMB.EXACT(“grand mal epilepsy”) or MESH.EXACT(“Epilepsy, Tonic-Clonic”) |
| S3 | TI,AB(absenteeism OR presenteeism OR ((productivity OR productive OR sick OR work OR sickness OR disability OR employee OR employer) NEAR/3 (limitations OR impact OR day OR days OR leave OR absence OR absences OR incapacity OR loss OR lost OR losing)) OR “loss-of-work” OR (cost* NEAR/3 (indirect OR informal OR economic OR societal OR intangible OR caregiver))) OR EMB.EXACT(“productivity” OR “medical leave” OR “presenteeism” OR “absenteeism”) OR EMB.EXACT.EXPLODE(“salary and fringe benefit”) OR MESH.EXACT(“Employer Health Costs” OR “Efficiency” OR “Presenteeism” OR “Absenteeism”) OR MESH.EXACT.EXPLODE(“Salaries and Fringe Benefits”) OR EMB.EXACT(“work productivity and activity impairment questionnaire”) OR EMB.EXACT(“work productivity and activity impairment questionnaire: general health”) OR TI,AB(work productivity and activity impairment) |
| S4 | TI,AB(((“short form” OR shortform OR SF) NEAR/1 (six OR 6 OR eight OR 8 OR twelve OR 12 OR sixteen OR 16 OR twenty OR 20 OR “thirty six” OR 36)) OR ((quality OR disability) NEAR/1 adjusted) OR “quality of life” OR qol OR hrqol OR qaly OR qalys OR daly OR dalys OR “life year” OR “life years” OR ((health OR healthy) NEAR/1 (year OR years OR status OR indicator OR indicators)) OR QLS OR “well being” OR wellbeing OR QWB OR “Nottingham health profile[*2]” OR “sickness impact profile” OR utility OR utilities OR preference OR preferences OR prefer OR disutility OR rosser OR “willingness to pay” OR WTP OR “standard gambl[*4]” OR “trade off” OR tradeoff OR “trade-off” OR hui1 OR hui2 OR hui3 OR euroqol OR “euro qol” OR eq5d OR “eq 5d” OR euroqual OR “euro qual” OR (European NEAR/1 (qol OR quality)) OR “duke health profile” OR “functional status questionnaire” OR “roportio coop functional health assessment*” OR ((illness OR health) NEAR/1 (state[*1]) OR multiattribute* OR “multi attribute*”)) OR TI,AB(Qualitative research) OR TI,AB(Focus groups) OR TI,AB(Semi-structured interview) OR TI,AB(Nursing methodology research) OR TI,AB(Constant comparative method) OR TI,AB(Purposive sampling) OR TI,AB(Phenomenological research) OR TI,AB(Life experiences) OR TI,AB(Grounded research) OR TI,AB(Qualitative analysis) OR TI,AB(Beck depression inventory II) OR TI,AB(Impact of epilepsy scale) OR TI,AB(Impact of epilepsy scale) OR TI,AB(Quality of life in newly diagnosed epilepsy) OR TI,AB(Quality of life in epilepsy inventory-89) OR TI,AB(Quality of life in epilepsy inventory-31) OR TI,AB(Quality of life in epilepsy inventory-10) OR TI,AB(Washington psychosocial seizure inventory) OR EMB.EXACT(“Beck depression inventory”) OR EMB.EXACT(“Center for epidemiological studies depression scale”) OR EMB.EXACT(“Hospital anxiety and depression scale”) OR EMB.EXACT(“Rosenberg self-esteem scale”) OR TI,AB(hrql) OR EMB.EXACT(“quality-adjusted life year” OR “roportion health profile” OR “sickness impact profile” OR “patient satisfaction” OR “attitude to health” OR “patient-reported outcome”) OR EMB.EXACT.EXPLODE(“health status indicator” OR “quality of life”) OR MESH.EXACT(“Value of Life” OR “Quality of Life” OR “Quality-Adjusted Life Years” OR “Attitude to Health” OR “Patient Reported Outcome Measures” OR “Patient Outcome Assessment”) OR MESH.EXACT.EXPLODE(“Health Status Indicators” OR “Patient Satisfaction”) OR EMB.EXACT.EXPLODE(“Quality of Life in Childhood Epilepsy Questionnaire”) OR TI,AB(Quality of Life in Childhood Epilepsy) OR TI,AB(QOLCE) OR TI,AB(Health-related Quality of Life Measure for Children with Epilepsy) OR TI,AB(CHEQOL) OR TI,AB(Quality of Life Questionnaire in Children with Epilepsy) OR TI,AB(QOLIED) OR TI,AB(KINDLR) OR TI,AB(Pediatric Quality of Life Inventory) OR TI,AB(PedsQL) OR EMB.EXACT.EXPLODE(“Pediatric Quality of Life Inventory”) OR EMB.EXACT.EXPLODE(“Epworth sleepiness scale”) OR TI,AB(Epworth sleepiness scale) OR TI,AB(Epilepsy questionnaire score) OR EMB.EXACT.EXPLODE(“Generalized anxiety disorder-7”) OR TI,AB(Generalized anxiety disorder-7) OR EMB.EXACT.EXPLODE(“Profile of mood states”) OR TI,AB(Profile of mood states) OR TI,AB(Neurological disorders depression inventory for epilepsy) OR TI,AB(NDDI-E) OR TI,AB(Brief irritability test) OR TI,AB(Questionnaire for irritability) OR TI,AB(Irritability Questionnaire) OR TI,AB(“I-epi”) OR EMB.EXACT.EXPLODE(“neuropsychiatric inventory”) OR TI,AB(“NPI”) OR TI,AB(Epilepsy Anxiety Survey Instrument) OR TI,AB(“EASI”) OR TI,AB(“brEASI”) |
| S5 | EMB.EXACT(“case study” OR “case report” OR “abstract report” OR “note”) OR DTYPE(“Historical Article” OR “Editorial” OR “Note” OR Comment OR News OR “Newspaper Article” OR Review OR “Conference Abstract”) OR TI,AB(“case study” or “case studies” OR “case report” OR “case reports”) |
| S6 | (S1 OR S2) AND (S3 OR S4) |
| S7 | S6 NOT S5 |
| S8 | S7 AND PD(>2011) NOT DTYPE(“Conference abstract”) |
| S9 | S6 AND DTYPE(“Conference abstract”) AND PD(>2018) |

Table S2. PICOS Eligibility Criteria

|  | **Inclusion Criteria** | **Exclusion Criteria** |
| --- | --- | --- |
| Patient Population | Adult patients with focal epilepsy^a^  Adult patients with primary generalized tonic-clonic seizures^a^  Caregivers of adult patients with focal epilepsy^a^  Caregivers of adult patients with generalized tonic-clonic seizures^a^ | Adult patients with epilepsy or seizure type not listed in inclusion criteria  Caregivers of adult patients with epilepsy or seizure type not listed in inclusion criteria |
| Intervention | Any or none | Not applicable |
| Comparator | Any or none | Not applicable |
| Outcomes | Humanistic burden:  Patient-reported or caregiver reported outcomes or utilities  Qualitative evaluation of quality of life, function and activities of daily living, and other humanistic burden outcomes  Economic burden:  Productivity loss for patients and/or caregivers  Caregiver costs  Societal costs | Not applicable |
| Study Design | Interventional trials (randomized or single arm)  Observational studies  Qualitative research studies  Systematic reviews (for identification of primary studies only)^b^ | Editorials, notes, or commentary  Non-systematic reviews  Case reports/case series |
| Other | English language  Articles and conference abstracts indexed in Embase and MEDLINE and published 2012 or later  Sample size >70 population of interest^c^  Conference abstracts presented at European Epilepsy Congress^d^ | Non-English language |
| ^a^ Study populations comprising >50% of the population of interest were included.  ^b^ Bibliographies of systematic reviews were scanned for studies of interest.  ^c^ Criteria did not apply for qualitative studies.  ^d^ Relevant abstracts were hand-searched as this conference is not indexed in Embase. | | |

Table S3. Patient-Reported Outcome Instruments in Focal Epilepsy

| **Full Name** | **Short Name** | **Measures** | **Scale structure** | **Score range** | **Score interpretation** | **Studies** |
| --- | --- | --- | --- | --- | --- | --- |
| **Symptom Burden Due to Epilepsy or Anti-Seizure Medications** | | | | | |  |
| **Epilepsy-Specific** |  |  |  |  |  |  |
| Liverpool Adverse Events Profile | LAEP | Patient perception of AEs from antiepileptic drugs | 19 items | 19-76 | Higher score indicates greater burden due to AEs | 8 |
| Liverpool Seizure Severity Scale | LSSS | Severity of seizures in PWE | 12 items across 1 dimension | 0-100 | Higher scores indicate greater perceived seizure severity | 4 |
| Sudden Unexplained Death Risk in Epilepsy | SUDEP-7 | Sudden unexplained death in epilepsy risk in PWE | 7 items | 0-12 | Higher scores indicate greater risk of sudden unexplained death | 1 |
| **Generic** |  |  |  |  |  |  |
| International Restless Legs Syndrome Study Group Severity Scale | IRLSSG severity scale | Severity of restless leg syndrome | 10 questions | 0-40 | Higher scores indicate worse severity. Categories are mild (1-10), moderate (11-20), severe (21-30), and very severe (31-40) | 1 |
| **Functional Status** | | | | | | |
| **Epilepsy-Specific** |  |  |  |  |  |  |
| Epilepsy Self-Efficacy Scale | ESES | Perceived self-efficacy in performing tasks related to epilepsy management | 18 items | 0-180 | Higher scores indicate higher level of confidence in ability to manage epilepsy | 1 |
| **Generic** |  |  |  |  |  |  |
| A-B Neuropsychological Assessment Schedule | ABNAS | Objective impairment | NR | 0-72 | Higher scores indicate greater symptomatology. Score of 15 indicates significant subjective symptoms | 1 |
| Difficulties in Emotion Regulation Scale | DERS | Emotion regulation | 36 items across 6 subscales | 36-180 | Higher scores indicate more difficulty in emotion regulation | 1 |
| Dissociative Experiences Scale | DES | Dissociation | 28 items across 3 subcategories | 0-100 | Higher scores indicate higher levels of dissociation.  Scores 20-30 indicate possible PTSD or DDNOS. Scores >30 indicates possible DID. | 1 |
| Female Sexual Function Index | FSFI | Sexual function | 19 items | 2-36 | Higher scores indicate better sexual function | 1 |
| Patient Global Impression of Change | PGIC | Changes in functioning over time | 7-point scale | 1-7 | Scores range from 1 (very much improved) to 7 (very much worse) | 1 |
| Self-Report Social Adjustment Scale | SAS | Social adjustment performance | 42 items across 5 domains | NR | Higher scores indicate more severe maladjustment | 2 |
| Side Effects and life Satisfaction Inventory | SEALS | Effects of anti-epileptic drugs on cognition | 38 items across 5 domains | NR | Higher scores indicate worse effects | 1 |
| Sheehan Disability Scale | SDS | Impairment in functioning | 3 items | 0-30 | Higher scores indicate higher functional impairment | 1 |
| **Mental Health** | | | | | | |
| **Epilepsy-Specific** |  |  |  |  |  |  |
| Neurological Disorder Depression Inventory for Epilepsy | NDDI-E | Depression | 6 items | 6-24 | Higher score corresponds to increased depressive symptoms.  Scores of ≥12 are suggestive of MDD, and ≥15 considered the clinical cutoff consistent with MDD diagnosis | 26 |
| **Generic** |  |  |  |  |  |  |
| Beck Anxiety Inventory | BAI | Anxiety | 21 items | 0-63 | Higher scores indicate more severe anxiety. Severity categorized as minimal (0-7), mild (8-15), moderate (16-25), and severe (30-63). | 5 |
| Beck Depression Inventory | BDI | Depression | 21 items | 0-63 | Higher scores indicate more severe depression. Scores of >9 suggestive of depression, with severity categorized as mild (10-18), moderate (19-29), and severe (≥30) | 29 |
| Center for Epidemiological Studies Depression Scale | CES-D | Symptoms of depression over past week | 20 items | 0-60 | Higher scores indicate greater impairment | 1 |
| (Electronic-) Columbia-Suicide Severity Rating Scale | (E-) CSSRS | Suicidal ideation and behavior | 4 versions of scale; 10 categories (yes/no) | N/A | Suicidal ideation: “yes” to categories 1-5  Suicidal behavior: “yes” to categories 6-10 | 3 |
| General Anxiety Disorder 7-item | GAD-7 | Generalized anxiety disorder | 7 items | 0-21 | Higher score indicates more intense anxiety.  Score ≥7 suggestive of general anxiety disorder.  Score categories include mild (5-9), moderate (10-14), and severe anxiety (≥15) | 13 |
| Goldberg Anxiety Scale | GADS | Anxiety | 18 items over 2 symptoms scales | 0-18 | Higher scores indicate greater anxiety. | 1 |
| Hamilton Anxiety Scale | HAM-A | Anxiety | 14 items | 0-56 | Higher scores indicate more severe anxiety, categorized as mild (<17), mild-to-moderate (18-24), and moderate-to-severe (25-30) | 1 |
| Hamilton Depression Scale | HAM-D (or HDRS) | Depression | 17 items | 0-54 | Higher scores indicate more severe depression,  categorized as normal (0-6), mild (7-17), moderate (18-24), severe (19-22), and very severe (≥24) | 2 |
| Hospital Anxiety and Depression Scale | HADS | Anxiety and depression | 7 items each measuring anxiety or depression | 0-21 | Higher scores indicate higher levels of anxiety or depression.  Score ≥8 defined as presence of symptoms | 22 |
| Inventory of Depressive Symptomatology Self-Report | IDS-SR | Depression | 30 items | 0-84 | Higher scores indicate greater symptomatology | 1 |
| Montgomery-Åsberg Depression Rating Scale | MADRS | Depression | 10 items | 0-60 | Higher scores indicate worse depressive symptoms | 2 |
| Neuropsychiatric Inventory-Questionnaire | NPI-Q | Neuropsychiatric symptomatology | 12 domains | 0-36 (severity);  0-60 (distress) | Higher scores indicate worse symptomatology | 1 |
| Obsessive-Compulsive Inventory-Revised | OCI-R | Distress associated with obsessions and compulsions | 18 items over 6 categories | 0-72 | Higher scores indicate higher level of obsessive-compulsive behavior | 1 |
| Patient Health Questionnaire 9-Item | PHQ-9 | Depressive symptoms during previous 2 weeks | 9 -items | 0-27 | Higher scores indicate higher risk of developing depression, categorized as minimal (0-4), mild (5-9), moderate (10-14), moderately severe (15-19), and severe (20-27) | 8 |
| Perceived Stress Scale | PSS | Stress levels over past month | 14-items (shortened versions available) | 0-40 | Higher score indicates higher perceived stress | 3 |
| Profile of Mood States Short-Form | POMS-SF | Psychological distress | 37 items over 6 dimensions | NR | Higher scores indicate increased distress | 1 |
| Temperament Evaluation of Memphis, Pisa, Paris, and San Diego | TEMPS-A | Affective temperaments | 109 items (men)/ 110 items (women) across 5 subdimensions | 0-109/ 110 | Higher scores indicate greater affect | 1 |
| Toronto Alexithymia Scale | TAS-20 | Alexithymia | 20 items across 3 subscales | 20-100 | Higher scores indicate greater impairment, categorized as no alexithymia (0-51), possible alexithymia (52-60), and alexithymia present (61-100) | 1 |
| State Trait Anxiety Scale | STAI | State and trait anxiety | 40 items across 2 subscales | 20-80 | Higher scores indicate greater anxiety, and categorized as very low (<35), low (36-45), medium (46-55), high (56-65), and very high (>65) | 3 |
| World Health Organization Well-being Index | WHO-5 | Current mental wellbeing | 5 items | Raw: 0-25  Total: 4-100 | Higher scores indicate better wellbeing | 1 |
| Zung Self-Rating Anxiety and Depression Scale | SAS/SDS | Anxiety or depression | 40 items across 2 scales | 20-80 | Higher scores indicate more severe anxiety or depression | 2 |
| **Sleep** | | | | | | |
| **Epilepsy-specific** |  |  |  |  |  |  |
| None |  |  |  |  |  |  |
| **Generic** |  |  |  |  |  |  |
| Berlin Questionnaire | BQ | Risk of OSA syndrome | 10 questions in 3 categories | N/A | Categories evaluated separately; if ≥2 categories have positive results, OSA syndrome risk is high | 3 |
| Epworth Sleepiness Scale | ESS | Daytime sleepiness | 8 questions | 0-24 | Higher scores indicate greater severity of excessive daytimes sleepiness.  Score of ≥10 indicates excessive daytime sleepiness | 11 |
| Innsbruck REM Sleep Behavior Disorder Inventory | N/A | Potential REM sleep behavior disorder | 5-items | NR | A cut-off of 0.25 suggests potential REM sleep behavior disorder | 1 |
| Insomnia Severity Index | ISI | Sleep difficulties over previous 2 weeks | 7-tems (5-point scale) | 0-28 | Higher scores indicate more sleep difficulties.  Score >7 indicates insomnia. | 5 |
| Munich Chronotype Questionnaire | N/A | Sleep-wake pattern on working and non-working days | 17 items comprising 4 domains | N/A | Captures time for each item (eg, sleep onset on workdays) | 1 |
| Munich Parasomnia Scale | N/A | Lifetime prevalence and current frequency of parasomnias and nocturnal behaviors in adults | 21 items | N/A | Parasomnias and behaviors scored as yes/no | 1 |
| Pittsburgh Sleep Quality Index | PSQI | General sleep patterns | 18 questions comprising 7 subheadings | 0-21 | Higher scores indicate worse sleep quality. Score of ≥5 indicates poor sleep quality | 6 |
| Sleep Apnea Scale of the Sleep Disorder Questionnaire | SA/SDQ | Likelihood of OSA | 12 items | NR | In PWE, cutoffs ≥26 for women and ≥29 for men correlate with diagnosis of OSA | 1 |
| **Personality and Beliefs** | | | | | | |
| **Epilepsy-specific** |  |  |  |  |  |  |
| Irritability in Adult Patients with Epilepsy | I-Epi | Interictal irritability | 4 domains | 18-108 | Higher scores indicate more intense irritability | 1 |
| **Generic** |  |  |  |  |  |  |
| Brief Illness Perception Questionnaire | B-IPQ | Cognitive and emotional representation of illness | 9 items | 0-10 | Higher scores indicate a more threatening view of illness | 1 |
| Buss-Perry Aggression Questionnaire | BAQ | Aggression | 29 items | 29-145 | Higher scores indicate higher levels of aggression | 2 |
| Beliefs About Medicine – Self-report | BMQ-S | Beliefs about medicine | 2 5-item scales | 5-25 (per scale) | Higher scores indicate stronger beliefs | 1 |
| Centrality of Religiosity Scale | CRS-5 | Centrality, importance, and salience of a person’s religious meaning | 15 items | 1-5 | Higher scores indicate more centrality | 1 |
| Eysenck Personality Questionnaire-Revised | EPQ-RS | Neuroticism and extroversion | 2 12-item scales | 0-12 (per scale) | Higher scores indicate higher levels of neuroticism and extraversion | 1 |
| General Self-Efficacy Scale | GSES | Self-efficacy | 17 items | 17-85 | Higher scores indicate greater general self-efficacy | 1 |
| Index of Core Spiritual Experience | INSPIRIT-R | Religiosity and spirituality | 7 items | 7-28 | Higher scores indicate higher religiosity and spirituality | 1 |
| Irritability Questionnaire | IRQ | Irritability | 21 items | NR | Higher scores indicate greater irritability | 1 |
| Modified Overt Aggression Scale | MOAS | Verbal aggression, aggression against property, auto-aggression, and physical aggression | 4-items | 0-40 | Weighted score with higher scores indicating higher aggression | 1 |
| Personality Assessment Inventory | PAI | Psychological and interpersonal domains | 344 items across 11 core clinical domains | 0-100 | Raw scores transformed to T-scores.  Scores 50-70 considered within normal limits; scores >70 (or conservatively, ≥65) suggest underlying psychological distress. | 1 |
| Rosenberg Self-Esteem Scale | RSES | Self-worth | 10-items | 10-40 | Higher scores indicate better self-esteem | 2 |
| Work Self-Determination Index/ Work Extrinsic and Intrinsic Motivation scale | WSDI/ WEIMS | Level of work motivation | 18 items across 6 subscales | NR | Higher scores indicate higher work motivation | 1 |
| **Family and Social Support** | | | | | | |
| **Epilepsy-specific** |  |  |  |  |  |  |
| None |  |  |  |  |  |  |
| **Generic** |  |  |  |  |  |  |
| Family Adaptability, Partnership, Growth, Affection, and Resolve Scale | APGAR | Satisfaction with family support | 5 statements | 0-20 | Higher scores indicate greater satisfaction with family support | 3 |
| Family Adaptability and Cohesion Evaluation Scale III | FACES III | Family function | 20 items across 2 scales | 20-100 (10-50 per scale) | Higher scores indicate better family functioning | 1 |
| Family Adaptability and Cohesion Evaluation Scale IV | FACES IV | Family functioning | 62 items across 6 scales | NR | Higher scores indicate better family functioning | 1 |
| Multidimensional Scale of Perceived Social Support | MSPSS | Degree of social support from family, friends, and significant others | 12-items | 12-84 | Higher scores indicate greater perceived support. | 1 |
| Oslo 3 Item Scale of Social Support | OSSS-3 | Level of social support | 3 items | 3-14 | Higher scores indicate greater social support, categorized as low (3-8), moderate (9-11), and high (12-14) | 1 |
| Social Interaction Anxiety Scale | SIAS | Fear in social interaction situations (companion of SPS) | 20-items | 0-80 | Higher scores indicate worse severity of social anxiety symptoms | 1 |
| Social Phobia Scale | SPS | Fear during performance situations (companion of SIAS) | 20-items | 0-80 | Higher scores indicate worse severity of social anxiety symptoms | 1 |
| Social Support Rating Scale | SSRS | Degree of social support | 10 items | 12-66 | Higher scores indicate greater social support | 1 |
| **Stigma and Discrimination** | | | | | | |
| **Epilepsy-Specific** |  |  |  |  |  |  |
| Epilepsy Stigma Scale | ESS | Perceived stigma in PWE | 3 items | 0-3 | Score ≥1 indicates any perceived stigma. Score of 3 indicates perception of being severely stigmatized | 4 |
| Stigma Scale of Epilepsy | SSE | Degree of stigma perceived by adults in different contexts | 24 items comprising 5 domains | 0-100 | Higher score indicates increased perception of stigma | 5 |
| **Generic** |  |  |  |  |  |  |
| Discrimination and Stigma Scale | DISC-12 | Discrimination and support at work | 5 items (original DISC-12 utilizes 12 items) | 0-15 | Higher score indicates more experienced discrimination and stigma | 1 |
| Enacted Stigma | ES | Enacted stigma (i.e., instances of discrimination against individuals with a stigmatized condition) | 4 questions | N/A | Yes response to ≥1 question indicates enacted stigma | 1 |
| Jacoby’s Stigma Scale | JSS | Stigma | 3-items | 0-3 | Higher scores indicate greater sense of stigma | 1 |
| Revised Stigma Scale | RSS | Perceived stigma | 3 items | 0-9 | Higher scores indicate higher perceived stigma | 1 |
| **HRQOL** | | | | | | |
| **Epilepsy-Specific** |  |  |  |  |  |  |
| Epilepsy Surgery Inventory – 55 Items | ESI-55 | HRQOL | 55 items across 11 scales | 0-100 | Higher scores indicate better HRQOL | 3 |
| Performance, Sociodemographic aspects, Subjective evaluation/ estimation | PESOS | Epilepsy-specific HRQOL | 4 subscales | N/A | Higher scores indicates worse HRQOL | 1 |
| Quality of Life in Epilepsy Inventory | QOLIE-89 | HRQOL in epilepsy | 89 items across 17 measures | 0-100 | Higher scores indicate better HRQOL | 4 |
|  | QOLIE-31^a^ | HRQOL in epilepsy | 31 items across 7 scales | 0-100 | Higher scores indicate better HRQOL | 40 |
|  | QOLIE-10 | HRQOL in epilepsy | 10 items across 3 components | 0-100 | Higher scores indicate better HRQOL | 12 |
| Quality of Life in Epilepsy-Patient-Weighted | QOLIE-31-P | HRQOL and perceived burden in epilepsy | 38 items across 8 components | 0-100 | Higher scores indicate better HRQOL | 8 |
|  | QOLIE-10-P | HRQOL and perceived burden in epilepsy | 11 items across 3 components | 0-100 | Higher scores indicate better HRQOL | 1 |
| **Generic** |  |  |  |  |  |  |
| EQ-5D | N/A | HRQOL | 5 items across 5 dimensions | 0-100 | Higher scores indicate better HRQOL | 9 |
| Global QOL | N/A | HRQOL | 9 items across 2 categories | 0-10 | Higher scores indicate better HRQOL | 1 |
| Neuro-Quality of Life | NQOL | HRQOL in people with neurological disorders | 14 instruments | 8-40 per scale | Higher scores indicate worse symptoms. Raw scores can be converted into T-scores. | 1 |
| Patient-Reported Outcomes Measurement Information System | PROMIS | Health | Approximately 70 domains | 0-100 | Raw scores converted to T-score. Higher score indicates more of the measured concept (eg, more fatigue) | 2 |
| Short Form Health Survey | SF-12 | General health | 12 items across 8 domains | 0-100 | Higher scores indicate better health. Mean US population score is 50 points (SD: 10) | 1 |
|  | SF-36 | General health | 36 items across 8 domains | 0-100 | Higher scores indicate better health. Mean US population score is 50 points (SD: 10) | 2 |
|  | SF-6D | General health | 11 items across 6 dimensions | 0-100 | Higher scores indicate better health. Mean US population score is 50 points (SD: 10) | 1 |
| World Health Organization Quality of Life Questionnaire | WHOQOL-BREF/26 | HRQOL | 26 items across 4 domains | 0-100 | Higher scores indicate better HRQOL | 2 |

Abbreviation: AE, adverse event; ASM, anti-seizure medication; DDNOS, dissociative disorder not otherwise specified; DID, dissociative identify disorder; HRQOL, health-related quality of life; MDD, major depressive disorder; N/A, not applicable; NR, not reported; peds, pediatric; OSA, obstructive sleep apnea; PTSD, post-traumatic stress disorder; PWE, people with epilepsy.

^a^ Includes modified QOLIE-31.

Table S4. Studies Reporting Qualitative Results of Humanistic Burden of Focal Epilepsy and/or Primary Generalized Tonic-Clonic Seizures

| **Reference** | **Country** | **Population (N)** | **Evaluation Method** | **Qualitative Summary Results** | | | |
| --- | --- | --- | --- | --- | --- | --- | --- |
|  |  |  |  | **Management of Disease** | **Activities of Daily Living** | **Symptoms Summary** | **Other** |
| French 2019^71 a^ | NR | FE (62) | Prospective qualitative semi-structured interviews | - | Most frequent or disturbing functional impairments (%; level of disturbance^b^):   - Inability to drive (74%; 7.1) - Limited ability to work and/or go to school (61%; 6.7) - Limitations on leisure and social activities (58%; 6.3) - Memory loss (47%; 8.4) | Most frequent or disturbing symptoms (%; level of disturbance^b^)   - Twitching/ tremors (80%; 5.3) - Confusion (78%: 7.8) - Difficulty talking (75%; 8.1) - Impaired/ loss of consciousness (70%; 6.8) - Stiffening (65%; 5.4) - Déjà vu (62%; 5.1) - Difficulty remembering (60%; 8.5) - Dizziness/ light-headedness (58%; 6.4) | Similar disease experiences were reported across the different stages of illness, though heterogeneity did exist (specific heterogeneities not reported by authors) |
| Kuramochi 2020^72^ | Japan | FE (10) | Prospective qualitative semi-structured interviews at outpatient psychiatric clinic | - | - | - | Five overarching categories were defined: 1) self-stigma; 2) cognition regarding public stigma; 3) anxiety and distress; 4) strategies for treating epilepsy; 5) other items |
| Perzynski 2017^73^ | US | Adults with epilepsy, including FE and PGTCS (22) | Prospective qualitative focus group | Common barriers and facilitators to self-management of epilepsy included:   - Personal barriers: psychological barriers, frustrations with doctors and healthcare systems - Personal facilitators: seizure planning, social facilitators, positive activities - Community and family barriers: societal (stigma, lack of public knowledge, misinformation), transportation, lack of support and resources - Community and family facilitators: social support, public knowledge - Health service barriers: provider barriers, healthcare access, treatment barriers, disability policy - Health service facilitators: provider support, alternative methods and medicine | - | - | - |

Abbreviations: FE, focal epilepsy; NR, not reported; PGTCS, primary generalized tonic clonic seizures; US, United States.

^a^ Conference abstract: results were limited. ^b^ Disturbance rating scale ranged from 0 (not at all) to 10 (extremely).

**Table S5. Patient- and Caregiver-Reported Outcome Instruments in PGTCS**

| **Instrument** | **Reference** | **Population (N)** | **Mean Score (SD)** | **Comparator** | **P-value** |
| --- | --- | --- | --- | --- | --- |
| **Symptom burden** | | | | | |
| LSSS | Sheikh 2020^70^ | People with epilepsy, GTCS (192) | Mean (SD): 34.0 (33.3)  Median (IQR): 36.25 (0-65) | No GTCS | 0.025^a^ |
|  |  | People with epilepsy, no GTCS (187) | Mean (SD): 23.7 (24.2)  Median (IQR): 20 (0-45) | Reference | NA |
| **Functional status** | | | | | |
| SAS Total | Paiva 2020^37^ | People with JME, majority with GTCS (112) | 2.14 (0.75) | Controls | <0.01 |
|  |  | Healthy controls (61) | 1.62 (0.55) | Reference | NA |
| **Mental health** | | | | | |
| NDDI-E | Jasionis 2021^78^ | People with GGE, majority with GTCS (27) | Median (range): 9 (6–20) | Across groups | 0.133 |
|  |  | People with ETLE (29) | Median (range): 12 (6–20) | Across groups | 0.133 |
|  |  | People with TLE (25) | Median (range): 11 (6–24) | Across groups | 0.133 |
| GAD-7 | Sheikh 2020^70^ | People with epilepsy, GTCS (192) | Mean (SD): 9.5 (6.5)  Median (IQR): 9 (4.5–14) | No GTCS | 0.049^a^ |
|  |  | People with epilepsy, no GTCS (187) | Mean (SD): 7.6 (6.3)  Median (IQR): 7 (2–12) | Reference | NA |
| HADS – Depression | Ahmad 2021^79^ | People with GTCS (200) | N (%) with depression: 68 (34) | Controls | <0.01 |
|  |  | Non-Epilepsy controls (200) | N (%) with depression: 20 (10) | Reference | NA |
| HADS – Anxiety | Ahmad 2021^79^ | People with GTCS (200) | N (%) with anxiety: 84 (42) | Controls | <0.01 |
|  |  | Non-Epilepsy controls (200) | N (%) with anxiety: 18 (9) | Reference | NA |
| PHQ-9 | Sheikh 2020^70^ | People with epilepsy, GTCS (192) | Mean (SD): 9.5 (6.5)  Median (IQR): 9 (4.5-14) | No GTCS | 0.035^a^ |
|  |  | People with epilepsy, no GTCS (187) | Mean (SD): 7.6 (6.3)  Median (IQR): 7 (2-12) | Reference | NA |
| STAI – State Anxiety | Paiva 2020^37^ | People with JME, majority with GTCS (112) | 38.21 (9.36) | Controls | <0.01 |
|  |  | Healthy controls (61) | 33.13 (8.98) | Reference | NA |
| STAI – Trait Anxiety | Paiva 2020^37^ | People with JME, majority with GTCS (112) | 43.10 (9.89) | Controls | <0.01 |
|  |  | Healthy controls (61) | 33.37 (8.27) | Reference | NA |
| **HRQOL** | | | | | |
| QOLIE-31 Total | Jasionis 2021^78^ | People with GGE, majority with GTCS (27) | 65.78 (18.10) | Across groups | 0.201 |
|  |  | People with ETLE (29) | 61.03 (15.85) | Across groups | 0.201 |
|  |  | People with TLE (25) | 56.73 (18.61) | Across groups | 0.201 |
| QOLIE-31 Overall QoL | Yadav 2022^80^ | People with GTCS, monotherapy (50) | 59.78 (18.14) | Polytherapy | 0.01 |
|  |  | People with GTCS, polytherapy (50) | 37.58 (18.45) | Reference | NA |
| QOLIE-10 Total | Sheikh 2020^70^ | People with epilepsy, GTCS (192) | Mean (SD): 28.7 (8.9)  Median (IQR): 28 (22-35) | No GTCS | 0.028^a^ |
|  |  | People with epilepsy, no GTCS (187) | Mean (SD): 25.6 (8.9)  Median (IQR): 25 (19-32) | Reference | NA |

Abbreviations: ETLE, extratemporal lobe epilepsy; GAD-7, General Anxiety Disorder 7-item; GGE, idiopathic (genetic) generalized epilepsy; GTCS, generalized tonic-clonic seizures; HADS, Hospital Anxiety and Depression Scale; JME, juvenile myoclonic epilepsy; LSSS, Liverpool Seizure Severity Scale; NA, not applicable; NDDI-E, Neurological Disorder Depression Inventory for Epilepsy; PGTCS, primary generalized tonic-clonic seizures; PHQ-9, Patient Health Questionnaire 9-Item; QOLIE, Quality of Life in Epilepsy Inventory; SAS, Self-Report Social Adjustment Scale; STAI, State and Trait Anxiety Scale; TLE, temporal lobe epilepsy.

^a^ Difference between groups was not significant after adjustment for covariates.
